# The proto-oncogene SRC phosphorylates cGAS to inhibit an antitumor immune response

**DOI:** 10.1172/jci.insight.167270

**Published:** 2023-06-22

**Authors:** William Dunker, Shivam A. Zaver, Jose Mario Bello Pineda, Cameron J. Howard, Robert K. Bradley, Joshua J. Woodward

**Affiliations:** 1 Department of Microbiology and; 2 Medical Scientist Training Program, University of Washington, Seattle, Washington, USA.; 3 Public Health Sciences and Basic Sciences Divisions, Fred Hutchinson Cancer Center, Seattle, Washington, USA.; 4 Department of Genome Sciences, University of Washington, Seattle, Washington, USA.

**Keywords:** Immunology, Oncology, Cancer immunotherapy, Innate immunity

## Abstract

Cyclic GMP-AMP synthase (cGAS) is a DNA sensor and responsible for inducing an antitumor immune response. Recent studies reveal that cGAS is frequently inhibited in cancer, and therapeutic targets to promote antitumor cGAS function remain elusive. SRC is a proto-oncogene tyrosine kinase and is expressed at elevated levels in numerous cancers. Here, we demonstrate that SRC expression in primary and metastatic bladder cancer negatively correlates with innate immune gene expression and immune cell infiltration. We determine that SRC restricts cGAS signaling in human cell lines through SRC small molecule inhibitors, depletion, and overexpression. cGAS and SRC interact in cells and in vitro*,* while SRC directly inhibits cGAS enzymatic activity and DNA binding in a kinase-dependent manner. SRC phosphorylates cGAS, and inhibition of cGAS Y248 phosphorylation partially reduces SRC inhibition. Collectively, our study demonstrates that cGAS antitumor signaling is hindered by the proto-oncogene SRC and describes how cancer-associated proteins can regulate the innate immune system.

## Graphical abstract

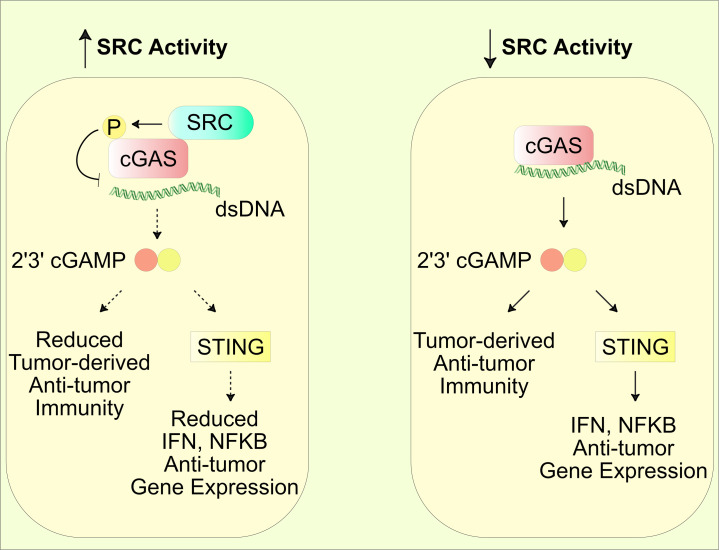

## Introduction

The activation and regulation of the cell intrinsic innate immune system is pivotal for the induction of an antimicrobial response to an infection, for restricting endogenous molecules from promoting an autoimmune state, and for initiating cell death following catastrophic cellular damage. Germline-encoded pattern recognition receptors (PRRs) are frontline defense sensors that recognize distinct microbial pathogen-associated molecular patterns (PAMPs) and damage-associated molecular patterns (DAMPs) to initiate an immune response (1). One PRR class includes nucleic acid sensors that recognize foreign and incorrectly localized or processed host DNA and RNA. dsDNA sensors include cyclic GMP-AMP synthase (cGAS), DNA-dependent activator of IFN regulatory factors (DAI), absent in melanoma 2 (AIM2), and DNA-dependent protein kinase (DNAPK), and activation of these receptors initiates various signaling cascades to generate an antimicrobial gene expression response (2–6). cGAS is the primary DNA sensor in most cell types and restricts a variety of pathogenic infections including viruses, bacteria, and fungi (7, 8). Following DNA binding in a sequence-independent manner, cGAS synthesizes the second messenger 2’3’ cyclic GMP-AMP (2’3’ cGAMP), which binds the protein stimulator of IFN genes (STING) on the endoplasmic reticulum (9–13). STING translocates to the Golgi and recruits TANK-binding kinase 1 (TBK1), which in turn activates the transcription factor complexes IFN regulatory factor (IRF) and NF-κB, inducing their nuclear translocation to stimulate IFN and proinflammatory cytokine expression, respectively.

Although the cGAS-STING pathway is canonically investigated in the context of infection, recent findings demonstrate that it can be activated by host DNA to impact cellular homeostasis independently of pathogens. Tight regulation of cGAS and endogenous dsDNA localization is essential for preventing cGAS activation of an aberrant immune response that contributes to various diseases, including autoimmune disorders such as systemic lupus erythematosus and Aicardi-Goutieres syndrome (14–18). Several cGAS regulatory mechanisms have been identified that inhibit the cGAS-STING pathway, including protein-protein interaction with BECN1 to limit 2’3’ cGAMP production and deSUMOylation by SUMO-specific peptidase 2 to induce cGAS degradation (19, 20). Furthermore, cGAS serine phosphorylation is a major inhibitory mechanism that restricts cGAS recognition of host DNA. AKT phosphorylation of cGAS reduces its enzymatic function, while phosphorylation by cyclin dependent kinase 1–cyclin B (CDK1–cyclin B) restricts cGAS activity during mitotic entry, and Aurora kinase B hyperphosphorylation of cGAS prevents recognition of chromatin during mitosis (21–23). Since cGAS is associated with numerous diseases, the identification of other cGAS regulatory mechanisms will provide the foundation for developing novel therapeutics.

cGAS stimulation is further associated with the induction of antitumor immunity in a variety of cancer subtypes by inducing expression of inflammatory genes and secretion of their protein products to recruit immune cells while also inducing replicative senescence that collectively restricts tumor growth and promotes tumor clearance (24, 25). Indeed, tumor-derived 2’3’ cGAMP is transferred to myeloid and B cells to induce NK cell–mediated tumor cell killing (26). Furthermore, pharmacological activation of the cGAS-STING pathway induces an antitumor response, suggesting that targeting cGAS in chemotherapeutic regimens would enhance patient outcomes (27–30). Unfortunately, cGAS and STING are inhibited or downregulated in various cancers through epigenetic, hypoxia, and posttranslational modification mechanisms (31–33). Since cGAS-STING activation generates an antitumor immune response, we hypothesized that cancer-associated proteins hinder cGAS-STING signaling by inhibiting cGAS activity to promote cancer progression. The proto-oncogene nonreceptor tyrosine kinase SRC is involved in several cellular pathways that enhance cancer development, including differentiation, proliferation, survival, and motility (34). Importantly SRC expression and kinase activity are frequently elevated in various cancers — including lung, breast, and colon cancer — and correlates with increased tumor initiation, progression, and metastasis (35). Therefore, SRC is pharmacologically inhibited by FDA-approved ATP competitive inhibitors such as bosutinib and dasatinib in chemotherapeutic cocktails for hematological cancer (36). Since cGAS is frequently inhibited in cancer, further investigation is needed to identify cancer-associated proteins such as SRC that prevent cGAS activation and are amenable to therapeutic intervention to drive an antitumor immune response.

SRC phosphorylates a variety of intracellular proteins and as SRC is highly expressed in cancer, it is likely that SRC also impacts other pathways that are involved in cancer biology such as nucleic acid sensing. Therefore, we sought to investigate whether SRC restricts cGAS activity through phosphorylation to limit an immune response. Here, we use publicly available data from The Cancer Genome Atlas (TCGA) to demonstrate that high *SRC* expression correlates with reduced immune gene expression and immune cell abundance in primary and metastatic bladder cancer. Furthermore, SRC restricts a dsDNA-dependent IFN and NF-κB immune response in lung adenocarcinoma, acute monocytic leukemia, and immortalized kidney cell lines. SRC inhibition enhances a cGAS-dependent immune response while SRC overexpression inhibits it in an SRC-kinase dependent manner. We further demonstrate that cGAS and SRC interact with each other in cells and in vitro, while SRC restricts cGAS enzymatic activity to synthesize 2’3’ cGAMP, potentially due to reduced cGAS DNA binding. Finally, we show that SRC directly phosphorylates cGAS, and genetically preventing phosphorylation at cGAS Y248 partially reduces the ability of SRC to restrict cGAS signaling. Collectively, our study describes how the proto-oncogene SRC regulates the DNA sensor cGAS and demonstrates how cancer-associated proteins can impact antitumor innate immune sensing.

## Results

### SRC expression correlates with reduced immune response in primary and metastatic cancer.

**Figure 1:**
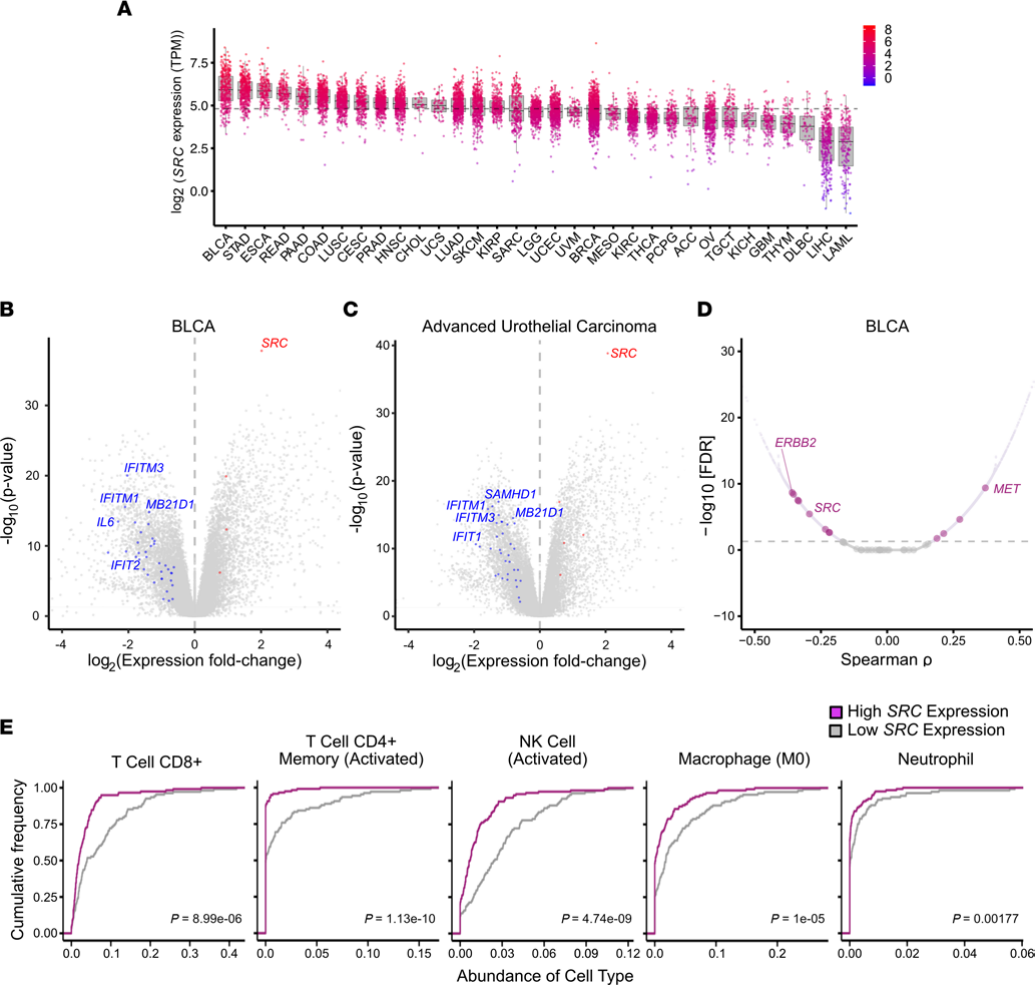
Reduced innate immune gene expression and immune cell abundance correlates with elevated SRC expression in primary and metastatic bladder cancer. (**A**) *SRC* expression in primary tumors from TCGA. Expression levels depicted in transcripts per million (TPM). Dashed horizontal line represents overall median expression. (**B**) *SRC* correlation volcano gene expression plot in primary BLCA. Colored dots represent Type I IFN genes with significant negative (blue) or positive (red) correlation. High (upper 66th percentile) versus low (lower 33rd percentile) *SRC* expressing samples were analyzed. (**C**) Volcano plot, similar to **B** in metastatic advanced urothelial carcinoma. (**D**) Correlation plot between oncogene expression in BLCA and normalized Type I IFN gene expression. Dashed horizontal line represents 5% FDR threshold. Statistical significance determined using Spearman correlation followed by multiple-hypothesis correction. (**E**) CIBERSORT analysis of immune cell abundance in BLCA tumors. High (upper 66th percentile) versus low (lower 33rd percentile) *SRC* expressing samples were analyzed. Statistical significance determined using 2-sided Mann-Whitney *U* test.

SRC is frequently highly expressed in certain cancers and is associated with enhanced cancer development (35). In addition, antitumor cGAS-STING signaling is typically reduced or inhibited in cancer to promote tumor progression (31–33). Therefore, we hypothesized that cancer types with elevated SRC expression levels would correlate with a reduced immune response due to inhibited cGAS DNA sensing. To first test this hypothesis, we used the TCGA database to analyze *SRC* expression in different primary cancer types. Urothelial Bladder Carcinoma (BLCA) expressed the highest *SRC* levels across the 33 cancer types analyzed (Figure 1A). Importantly, *SRC* was expressed at higher levels in tumor cohorts as compared with normal samples in several of the cancer types, including BLCA (Supplemental Figure 1A; supplemental material available online with this article; https://doi.org/10.1172/jci.insight.167270DS1). Since BLCA expressed the highest levels of *SRC*, we asked if *SRC* negatively correlates with Type I IFN gene expression in these cancers. Interestingly, gene expression analysis revealed that high *SRC* levels correlated with reduced expression of several immune-related genes, including the IFN stimulated genes (ISGs) *cGAS* and IFN induced protein with tetratricopeptide repeats 2 (*IFIT2*), and the NF-κB–transcribed gene *IL6* (Figure 1B and Supplemental Table 1). Furthermore, *SRC* negatively correlated with immune gene expression in metastatic advanced urothelial carcinoma tumors, suggesting that SRC may be involved in regulating immune signaling in various cancer stages (Figure 1C). Importantly, we also determined that *SRC* expression did not inversely correlate with immune genes by random chance. An unbiased correlation analysis, followed by multiple hypothesis correction of bladder cancer-associated oncogenes and Type I IFN–related genes, revealed that the *SRC-*immune gene expression correlation is substantial (Figure 1D). Our findings were further strengthened as ERB-B2 receptor tyrosine kinase 2 (*ERBB2*) also negatively correlated and is known to inhibit cGAS-STING signaling (Supplemental Figure 1B and Supplemental Table 2) (33). In addition, hepatocyte growth factor receptor (*MET*) positively correlated with Type I IFN–related genes, demonstrating that not all oncogenes were associated with reduced immune signaling (Supplemental Table 3).

Given that elevated SRC expression would inhibit cGAS signaling and reduce an antitumor immune response, we hypothesized that high SRC levels would correlate with reduced immune cell infiltration in tumors. To test this hypothesis, we analyzed the immune landscape in high versus low *SRC-*expressing bladder cancer tumors using CIBERSORT (37, 38). Intriguingly, high *SRC* expression was associated with reduced innate and adaptive immune cell abundance, including CD8^+^ T cell, NK cell, and macrophages (Figure 1E and Supplemental Figure 1C). In addition, there was a reduction in inflamed CD8^+^ T cells by IHC in high SRC–expressing advanced urothelial carcinoma tumors (Supplemental Figure 1D) (39). Interestingly, we found that elevated *SRC* and *cGAS* expression levels did not correlate with reduced and increased survival, respectively, although increased immune gene expression did correlate with enhanced survival in BLCA (Supplemental Figure 1E). In addition, *ERBB2* expression did not correlate with BLCA survival even though its expression negatively correlated with immune gene expression, suggesting that oncogene expression may not be an ideal indicator of patient outcomes. These data demonstrate that SRC negatively correlated with immune gene expression and immune cell infiltration in primary and metastatic tumors, suggesting that SRC may be regulating cGAS sensing as a means of inhibiting an antitumor immune response.

### SRC restricts a cGAS-dependent dsDNA immune response in various cell types.

SRC phosphorylates a variety of proteins to impact different pathways. To determine if SRC regulates a cGAS-dependent DNA-sensing immune response, we first validated a cellular coculturing method to analyze cGAS signaling without potential interference from SRC overexpression. The cyclic dinucleotide 2’3’ cGAMP produced in donor cells is transferred to recipient L929–IFN–stimulated response element–luciferase (L929-ISRE-LUC) reporter cells via connexins in gap junctions to induce ISRE-LUC expression (40, 41). We verified that various cyclic dinucleotides including 2’3’ cGAMP were transferred from HEK293T cells transfected with different cyclic dinucleotide cyclases into L929-ISRE-LUC cells by the induction of an ISRE response (Supplemental Figure 2A). Furthermore, transfection of cyclic dinucleotides directly into L929-ISRE-LUC cells induced ISRE activity, demonstrating that they were immunostimulatory (Supplemental Figure 2B). We also validated that 2’3’ cGAMP was required to induce an ISRE response by transfecting HEK293T cells with cGAS and Poxin, a known cGAMP cleavage enzyme, and observed a loss of ISRE induction (Supplemental Figure 2C) (42). Finally, we confirmed that 2’3’ cGAMP was transferred via gap junctions by treating the cocultured cells with meclofenamic acid (MFA), a gap junction antagonist, and did not detect an ISRE response (Supplemental Figure 2D). Overall, these data demonstrate that 2’3’ cGAMP was transferred from DNA-transfected cells into cocultured L929-ISRE-LUC cells to induce ISRE activity and can be used to determine if SRC regulates a cGAS-dependent immune response.

**Figure 2:**
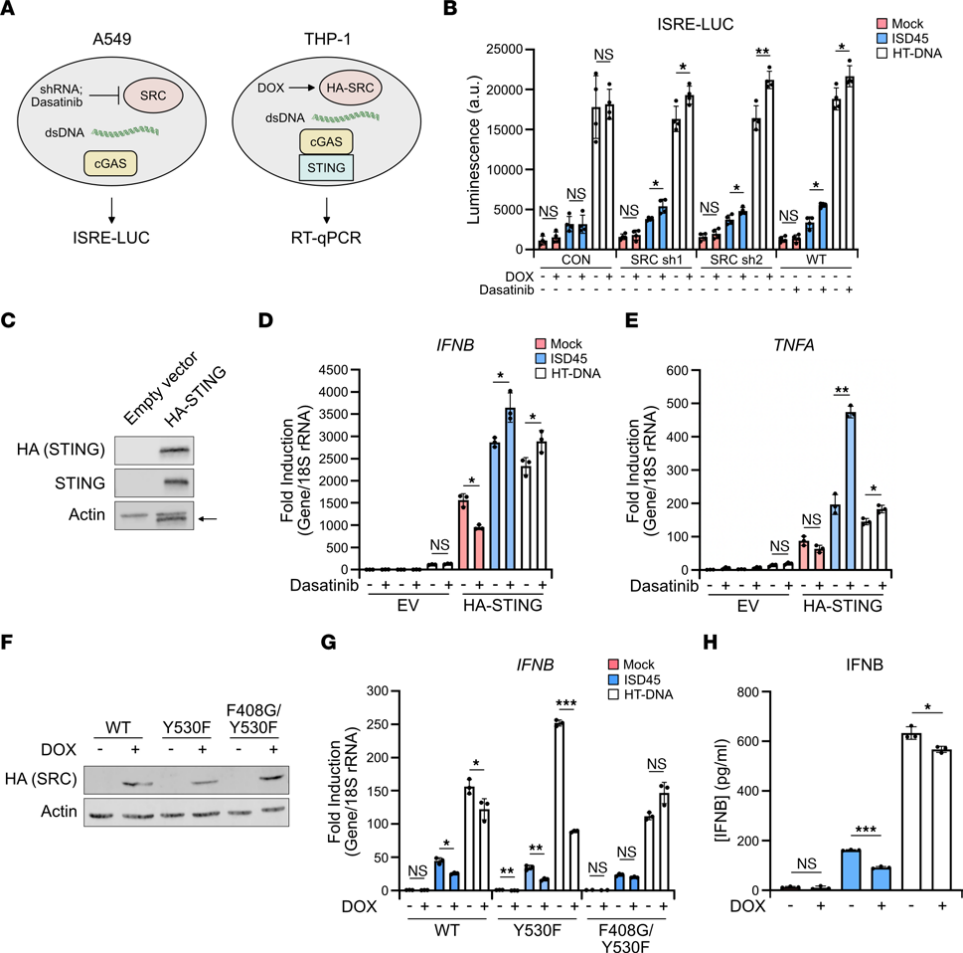
SRC restricts a DNA sensing immune response. (**A**) Schematic of SRC expression and activity manipulation in A549 and THP-1 cells. (**B**) Coculture of L929-ISRE-LUC cells with A549 cells transduced with DOX-inducible *SRC*-targeting shRNA or treated with 30 nM dasatinib followed by transfection of ISD45 or HT-DNA cGAS ligands for 6 hours. DOX was added for 72 hours and dasatinib for 48 hours (*n* = 4). (**C**) Western blot of A549 cells transfected with pcDNA3 empty vector (EV) or HA-STING pcDNA3. Arrow depicts nonspecific band. (**D**) qPCR analysis of *IFNB* levels from cells in **C** treated with vehicle or dasatinib for 48 hours followed by transfection of ISD45 or HT-DNA for 6 hours (*n* = 3). (**E**) qPCR of *TNFA* levels from cells in **D** (*n* = 3). (**F**) Western blot of THP-1 WT and mutant HA-SRC-tet-RB SB cells treated with 1 μg/mL DOX for 48 hours. (**G**) qPCR analysis of *IFNB* levels from cells in **F** treated with vehicle or DOX for 48 hours followed by transfection of ISD45 or HT-DNA for 6 hours (*n* = 3). (**H**) ELISA of IFNB levels from cells in **G**. Cells were transfected with ISD45 or HT-DNA for 24 hours (*n* = 3). All qPCR samples were normalized to 18S rRNA, and actin was used as a Western blot loading control. Student’s *t* test was used to determine statistical significance. Representative luciferase, qPCR, and ELISA shown. *n* = 3 (**B** and **G**) and *n* = 2 (**D**, **E**, and **H**).

A549 lung adenocarcinoma cells expressed cGAS and high levels of SRC compared with HEK293T and monocytic THP-1 cells, making them an ideal cell line to investigate SRC regulation of cGAS (Figure 2A and Supplemental Figure 3A). To determine if SRC regulates cGAS signaling, we treated A549 cells with the SRC small molecule inhibitor dasatinib. Furthermore, since dasatinib is a multikinase inhibitor, we validated that SRC restricts DNA sensing using doxycycline-inducible (DOX-inducible) shRNAs targeting *SRC*. Importantly dasatinib inhibited SRC activity in A549 cells, since autophosphorylation at SRC Y416 is lost after treatment, while shRNA induction significantly reduced *SRC* RNA levels (Supplemental Figure 3, B and C) (43). Following transfection of the cGAS ligands herring testes DNA (HT-DNA) and IFN stimulatory DNA (ISD45), and coculturing with L929-ISRE-LUC cells, A549 cells treated with dasatinib or DOX induced significantly higher ISRE activity as compared with mock-treated cells, suggesting that SRC inhibits cGAS signaling (Figure 2B). We further verified that the increase in ISRE activity was not due to dasatinib off-target effects, since saracatinib treatment, a different SRC inhibitor, also increased DNA sensing in A549 cells (Supplemental Figure 3D).

Given that A549 cells do not express STING, we next sought to determine whether STING expression would permit functional cGAS signaling and increased immune gene induction following SRC inhibition independent of a coculture. A549 cells were treated with dasatinib and transfected with a HA-tagged STING expression vector prior to cGAS ligand transfection and RNA isolation (Figure 2C). Importantly STING expression increased mRNA levels of the type I IFN, IFN-β1 (*IFNB*), in mock-treated cells as the vector itself activated cGAS, confirming a functional cGAS-STING pathway (Figure 2D). SRC inhibition induced significantly higher expression of various immune genes, including *IFNB*, the proinflammatory gene TNF (*TNFA*), and *IFIT2* following ligand transfection, demonstrating that SRC inhibition enhances a STING-dependent IFN and NF-κB immune response (Figure 2E and Supplemental Figure 3E).

Next, we sought to determine whether SRC overexpression impacts DNA signaling. Since transient transfection of DNA vectors induce cGAS activity, we generated stable DOX inducible HA-SRC sleeping beauty (SB) vectors that insert into the genome following selection (44). WT, kinase dead (KD) (K298M and F408G/Y530F), and kinase constituently active (Y530F) SRC mutants were produced to determine whether SRC kinase activity is responsible for inhibiting DNA sensing (45–49). Although the WT and SRC mutants were induced by DOX treatment in A549 cells, ISRE activity was not impacted following cGAS ligand transfection (Supplemental Figure 3, F and G). It is possible that the lack of ISRE induction is due to high levels of endogenous SRC in A549 cells where cGAS inhibition is already saturated. Therefore, we used THP-1 cells to overexpress HA-SRC as they expressed the cGAS-STING pathway and minimal SRC (Figure 2F). Importantly overexpression of WT and Y530F SRC in THP-1 cells significantly reduced IFNB and TNFA mRNA and cytokine induction following cGAS ligand transfection while the F408G/Y530F KD mutant did not impact their expression, suggesting that SRC overexpression restricted DNA sensing in a kinase-dependent manner (Figure 2, G and H, and Supplemental Figure 3, H and I). Furthermore, dasatinib treatment of THP-1 cells followed by cGAS ligand transfection did not impact *IFNB* expression, further demonstrating that SRC inhibition of cGAS depends on SRC and cGAS expression, as SRC is minimally expressed in THP-1 cells (Supplemental Figure 3J).

**Figure 3:**
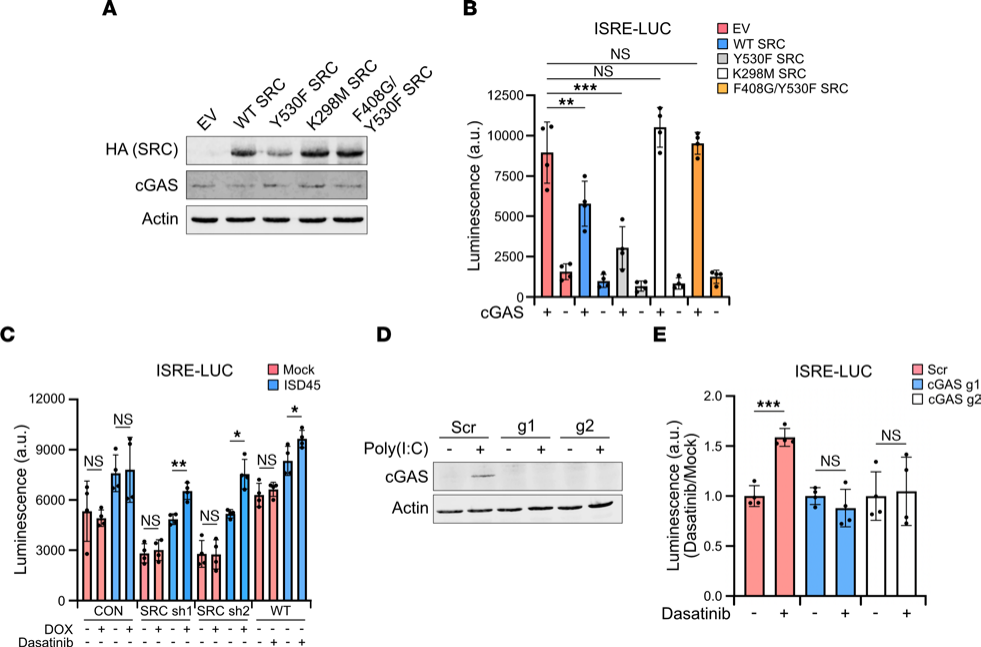
cGAS-dependent immune responses are hindered by SRC. (**A**) Western blot of HEK293T cells transfected with 75 ng cGAS-pcDNA3 and 925 ng of EV, WT SRC-HA pcDNA3, or mutant SRC-HA pcDNA3 for 24 hours. (**B**) Coculture of L929-ISRE-LUC cells with cells in **A** (*n* = 4). (**C**) Coculture of L929-ISRE-LUC cells with HEK293T cells transduced with DOX-inducible *SRC*-targeting shRNA or treated with 30 nM dasatinib followed by ISD45 transfection for 6 hours. DOX was added for 72 hours and dasatinib for 48 hours (*n* = 4). (**D**) Western blot of A549 cGAS–KO cells. Nontarget (Scr) guide RNA and 2 independent cGAS guide RNAs are shown. cGAS expression was induced by transfecting 1 μg/mL poly(I:C) for 16 hours. (**E**) Coculture of L929-ISRE-LUC cells with A549 Scr and cGAS-KO cells treated with vehicle (mock) or dasatinib (30nM) for 48 hours followed by transfection with HT-DNA for 6 hours. Mock luciferase levels for each cell line was set to 1 (*n* = 4). Actin was used as a Western blot loading control. Statistical significance tests: Student’s *t* test (**C** and **E**); 1-way ANOVA with Tukey test (**B**). Representative luciferase and qPCR shown. *n* = 3 (**B**) and *n* = 2 (**C** and **E**).

HEK293T cells express detectable SRC but not cGAS, allowing us to transfect DNA expression plasmids without activating an immune response (Supplemental Figure 3A). Therefore, to provide additional evidence that SRC overexpression inhibits cGAS signaling, we transfected HEK293T cells with WT, KD, and constituently active HA-SRC mutants with or without cGAS, followed by coculturing with L929-ISRE-LUC cells (Figure 3A). Consistent with SRC overexpression restricting cGAS signaling in THP-1 cells, WT and Y530F SRC significantly reduced ISRE activity in a cGAS-dependent manner (Figure 3B). Importantly both the K298M and F408G/Y530F KD mutants did not impact DNA sensing, validating a kinase-dependent inhibition. SRC kinase activity further restricted cGAS-dependent NF-κB signaling, as demonstrated through a NF-κB reporter LUC assay (Supplemental Figure 4A). We also analyzed murine *Ifnb* mRNA levels in the cocultured L929-ISRE-LUC cells as a different immune response measurement. Similar to ISRE-LUC induction, SRC restricted *Ifnb* expression in a kinase-dependent manner (Supplemental Figure 4B).

SRC is a member of the SRC family kinases (SFKs), a large family of conserved kinases that have redundant function and are inhibited by SRC inhibitors such as dasatinib, albeit with higher IC_50_ (50). Therefore, we sought to determine if SRC depletion and inhibition impacts cGAS signaling in HEK293T cells using dasatinib and DOX-inducible shRNAs. Dasatinib blocked SRC kinase activity while the shRNAs reduced *SRC* mRNA levels (Supplemental Figure 4, C and D). SRC depletion and inhibition significantly amplified cGAS-dependent ISRE activity following ISD45 transfection and coculturing with L929-ISRE-LUC cells, demonstrating that SRC inhibition and depletion increased cGAS signaling (Figure 3C). Importantly, SRC expression did not impact ISRE activity in response to 2’3’ cGAMP transfection, suggesting that inhibition occurs upstream of STING (Supplemental Figure 4E). We further validated that SRC restricts a DNA-dependent immune response in additional cancer cell lines. Pancreatic ASPC1 and 1.1B4 cells and colorectal HT29 cells were treated with dasatinib or a DOX-inducible shRNA, followed by HT-DNA transfection (Supplemental Figure 5A). Inhibition and depletion of SRC significantly elevated *IFNB* and *TNFA* mRNA induction compared with mock treatment, demonstrating that SRC restricts DNA sensing in a cell type–independent manner (Supplemental Figure 5, B and C). Intriguingly, Src inhibition in murine B16-BL6 melanoma cells did not increase *Ifnb* expression following DNA treatment but instead reduced it, suggesting that SRC restriction of cGAS is human specific (Supplemental Figure 5, D and E).

Lastly, to determine whether enhanced ISRE activity following SRC inhibition and coculturing is cGAS dependent, we generated cGAS CRISPR/Cas9 KOs in A549 cells. Given that cGAS is expressed at low levels under basil conditions and is an ISG, A549 cGAS–KO cells were transfected with the synthetic double-stranded RNA analog poly(I:C) to induce expression through the RIG-I–like receptor (RLR) RNA signaling pathway followed by Western blotting (51). cGAS was completely knocked out using 2 independent guide RNAs as compared with a nontarget scramble (Scr) guide, and we further validated the KOs by identifying the CRISPR/Cas9-induced mutations (Figure 3D). To determine whether SRC inhibits DNA sensing in a cGAS-dependent manner, we treated WT and KO cells with dasatinib, followed by cGAS ligand transfection and coculturing. While dasatinib enhanced ISRE activity in WT cells, the increase was completely abolished in cGAS-KO cells (Figure 3E). Collectively, these data demonstrate that SRC regulates a cGAS-dependent IFN and NF-κB gene expression response in multiple cell types.

### SRC interacts with cGAS and inhibits its enzymatic activity.

**Figure 4:**
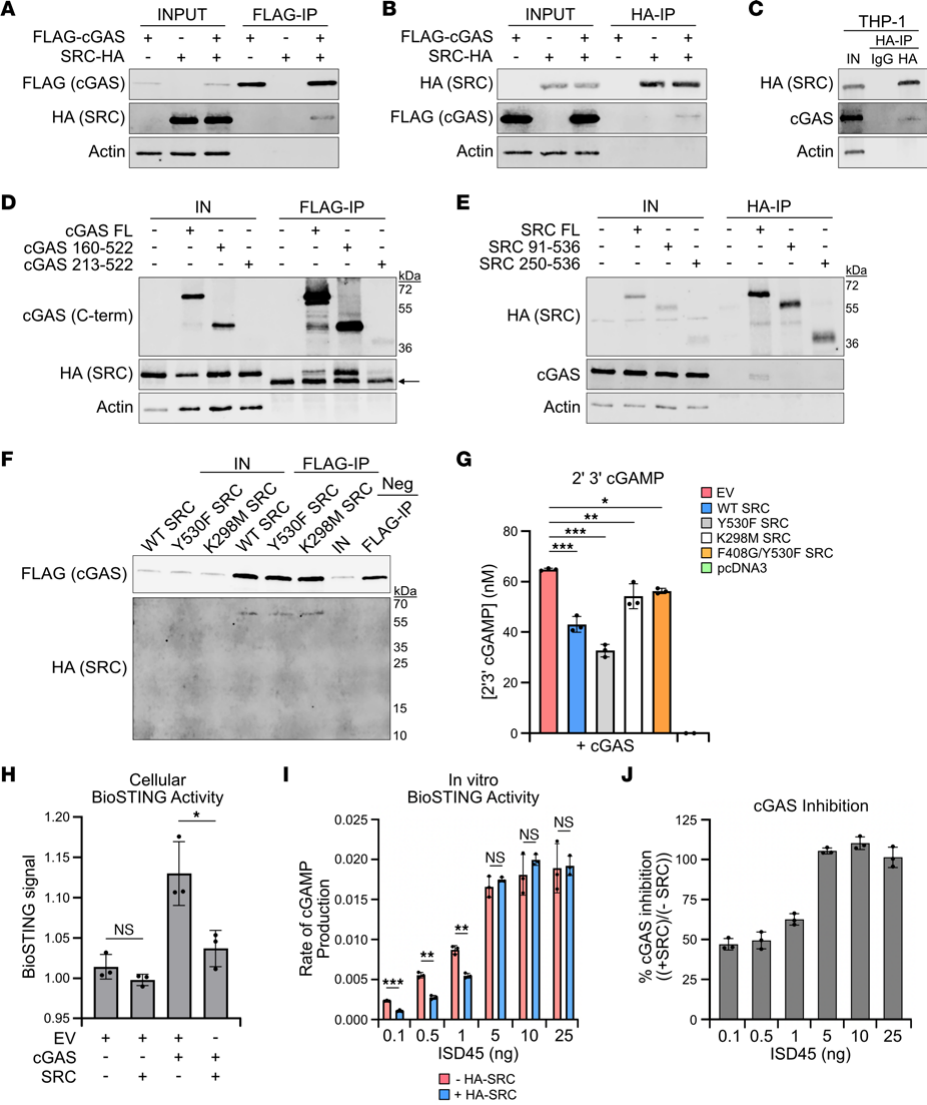
SRC directly interacts with cGAS and inhibits cGAS activation. (**A**) Western blot of HEK293T cells transfected with equal concentrations of EV, FLAG-cGAS pcDNA3, or SRC-HA pcDNA3 for 24 hours followed by FLAG IP. (**B**) Western blot, similar to **A** but HA IP. (**C**) Western blot of THP-1 WT HA-SRC pSB-tet-RB SB cells treated with DOX for 48 hours and poly(I:C) for 16 hours followed by a HA IP. (**D**) Western blot of HEK293T cells transfected with equal concentrations of EV, SRC-HA pcDNA3, or truncated FLAG-cGAS pcDNA3 for 24 hours followed by FLAG IP. Arrow depicts nonspecific band. (**E**) Western blot similar to **D** but with truncated SRC-HA pcDNA3 followed by HA IP. (**F**) Western blot of in vitro co-IP of FLAG-cGAS and WT or mutant HA-SRC. Equal concentrations of cGAS and SRC were incubated together followed by FLAG IP. A negative control HA-tagged protein (12 kDa) was used. (**G**) 2’3’ cGAMP EIA of HEK293T cells transfected with cGAS-pcDNA3 and EV, WT, or mutant SRC-HA pcDNA3 for 24 hours. pcDNA3 alone was used as a negative control (*n* = 3). (**H**) BioSTING FRET assay from HEK293T cells transfected with EV, cGAS pcDNA3, and SRC-HA pcDNA3 for 24 hours. Lysates were incubated with purified BioSTING, and activity was analyzed by FRET signal (*n* = 3). (**I**) BioSTING FRET assay using purified FLAG-cGAS phosphorylated by WT HA-SRC or mock treated prior to incubation with BioSTING and ISD45. Activity was analyzed by rate of 2’3’ cGAMP production as it relates to ISD45 concentration (*n* = 3). (**J**) Percentage of cGAS inhibition by SRC from data in **G**. Mock was set at 1, and HA-SRC treatment was compared with mock. Actin was used as a Western blot loading control. Statistical significance tests: Student’s *t* test (**H** and **I**); 1-way ANOVA with Tukey test (**G**). Representative assays are shown. *n* = 2 (**G** and **H**) and *n* = 3 (**I** and **J**).

Next, we investigated whether cGAS and SRC interact with each other, which could suggest a direct inhibitory mechanism. To determine if the proteins interact in cells, HEK293T cells were transfected with equal concentrations of empty vector (EV), FLAG-cGAS, or SRC-HA expression plasmids; subjected to a FLAG immunoprecipitation; and analyzed by Western blotting. cGAS was efficiently immunoprecipitated with and without SRC coexpression while SRC, but not the loading control actin, was pulled down in the immunoprecipitation (Figure 4A). Moreover, the reciprocal HA-IP revealed that FLAG-cGAS was also co-immunoprecipitated with SRC-HA (Figure 4B). To determine whether cGAS and SRC interact in THP-1 cells, a more physiologically relevant cell type, we treated inducible HA-SRC SB THP-1 cells with DOX, followed by a HA-IP. Importantly endogenous cGAS was co-immunoprecipitated with SRC, demonstrating that cGAS and SRC interact in various cell types (Figure 4C). Finally, to identify the cGAS and SRC domains that facilitate an interaction, we generated cGAS and SRC truncation plasmids. Full-length (FL) cGAS, loss of the N-terminal intrinsically disordered region (IDR) (aa 160–522), and only the Mab21 domain (aa 213–522) all interact with SRC (Figure 4D). Furthermore, only FL SRC interacted with cGAS, suggesting that N-terminal membrane anchoring may be required for the interaction (Figure 4E).

A direct interaction is required for SRC to phosphorylate cGAS. Therefore, to investigate whether cGAS and SRC directly interact, we purified FLAG-cGAS and HA-SRC from *E*. *coli* and HEK293T cells, respectively. We first validated that the purified proteins were active. FLAG-cGAS was incubated with ISD45, ATP, GTP, and purified BioSTING, a STING-based Förster resonance energy transfer (FRET) biosensor that measures 2’3’ cGAMP abundance by FRET signal (52) (Supplemental Figure 6A). Increasing concentrations of FLAG-cGAS enhanced BioSTING activity temporally due to 2’3’ cGAMP production, demonstrating that FLAG-cGAS is functional (Supplemental Figure 6B). Purified WT, K298M, and Y530F HA-SRC kinase activity were analyzed by in vitro kinase assays followed by Western blotting for autophosphorylation at Y416 (Supplemental Figure 6C). Importantly, WT and Y530F were phosphorylated but K298M was not, confirming that HA-SRC is active (Supplemental Figure 6D). To determine if cGAS directly interacts with HA-SRC and if the interaction is impacted by SRC kinase activity, equal concentrations of FLAG-cGAS and HA-SRC were incubated together, followed by a FLAG immunoprecipitation and analysis by Western blotting. WT, Y530F, and K298M SRC all interact with cGAS, while a HA-tagged negative control protein (12 kDa) did not interact (Figure 4F). Overall, these results suggest that SRC directly interacts with cGAS, and kinase activity does not affect binding.

Since SRC restricts cGAS signaling in a kinase-dependent manner, we hypothesized that SRC limits cGAS enzymatic ability to synthesize 2’3’ cGAMP, resulting in a reduced immune response. To determine if SRC restricts 2’3’ cGAMP production, we performed a 2’3’ cGAMP enzyme immunoassay on HEK293T cells expressing cGAS and the various SRC-HA constructs. Interestingly 2’3’ cGAMP levels were significantly reduced by WT and Y530F SRC but by not by the KD mutants (Figure 4G). We further analyzed 2’3’ cGAMP production by incubating cell lysates from HEK293T cells expressing cGAS and WT SRC-HA with purified BioSTING, followed by FRET analysis. BioSTING was activated in a cGAS-dependent manner, and activity was significantly reduced with SRC coexpression (Figure 4H). Finally, we determined whether SRC restricts cGAS activity in vitro by first subjecting purified FLAG-cGAS to an in vitro kinase assay using HA-SRC, followed by determination of 2’3 cGAMP production with BioSTING at various concentrations of ISD45. SRC significantly reduced the rate of 2’3’ cGAMP production in a DNA concentration-dependent manner (Figure 4I). At low DNA concentrations, SRC exhibited ~50% inhibition, while 2’3’ cGAMP production was indistinguishable at elevated DNA concentrations (Figure 4J). Analysis of the DNA required to achieve 50% of maximum cGAS activity revealed a 2-fold shift from 1.2 to 2.4 nanograms of ISD45. These data demonstrate that SRC interacted with cGAS and that SRC restricted cGAS enzymatic activity in a kinase-dependent manner, potentially through an altered affinity of cGAS for DNA.

### cGAS-DNA binding is reduced by SRC kinase activity.

**Figure 5:**
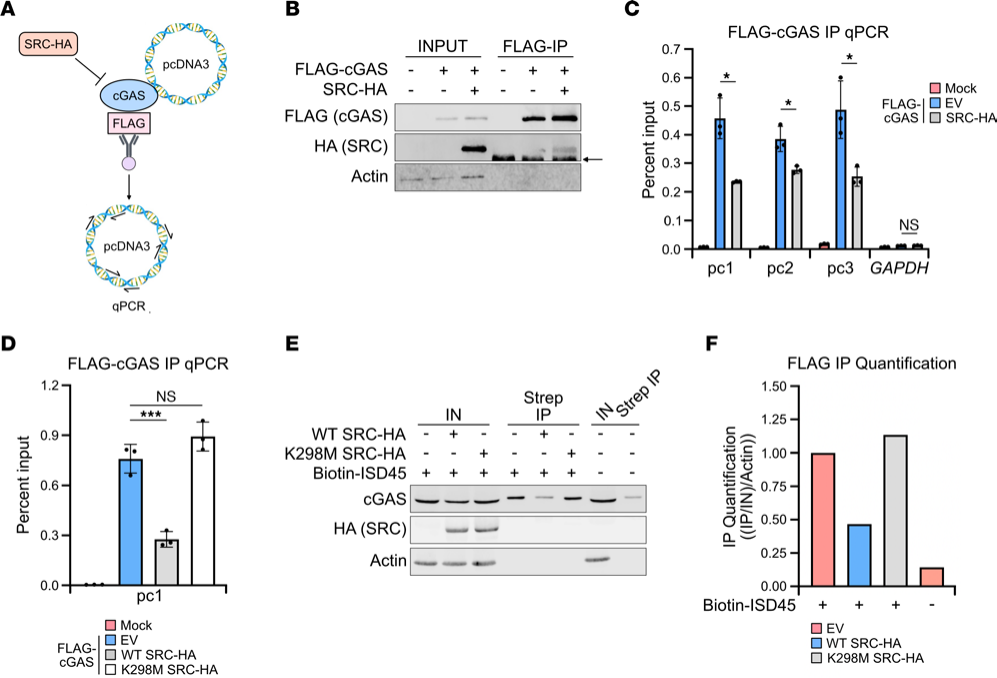
SRC kinase activity restricts cGAS DNA binding. (**A**) Schematic of FLAG-cGAS IP qPCR. HEK293T cells were transfected with EV, FLAG-cGAS pcDNA3, and SRC-HA pcDNA3 for 24 hours. Cells were crosslinked and subjected to FLAG IP coupled with qPCR analysis to detect bound pcDNA3 vector. (**B**) Western blot of FLAG-cGAS IP qPCR described in **A**. Arrow depicts nonspecific band. (**C**) IP qPCR analysis from FLAG-IP in **B**. Samples were quantified by percent input. Three different regions of pcDNA3 (pc1, pc2, pc3) were analyzed as well as nonspecific *GAPDH* (*n* = 3). (**D**) IP qPCR analysis of HEK293T cells transfected with FLAG-cGAS pcDNA3 and EV, WT, or K298M SRC-HA pcDNA3 followed by FLAG-cGAS (*n* = 3). (**E**) Western blot of HEK293T cells transfected with FLAG-cGAS pcDNA3 and EV, WT, or K298M SRC-HA pcDNA3 for 24 hours. Cells were then transfected with 4 μg/mL biotinylated or unmodified ISD45 for 2 hours followed by streptavidin (strep) IP. (**F**) Quantification of FLAG-cGAS enrichment from **E**. Actin was used as a Western blot loading control. Statistical significance tests: Student’s *t* test (**C**); 1-way ANOVA with Tukey test (**D**). Representative assays shown. *n* = 3 (**C**–**F**).

Inhibition of cGAS activity can occur through a variety of mechanisms. Restricting cGAS DNA binding is one such process and has frequently been identified as a means to limit cGAS signaling. cGAS phosphorylation by Aurora kinase B prevents chromatin binding during mitosis, while BAF nuclear assembly factor 1 (BAF) restricts nuclear cGAS activity by outcompeting cGAS for DNA binding (23, 53). To validate the in vitro kinetic observations and investigate whether SRC reduces cGAS DNA binding, we transfected HEK293T cells with FLAG-cGAS with or without SRC-HA, crosslinked cells, and performed a FLAG immunoprecipitation coupled with quantitative PCR (qPCR) (Figure 5A). Since plasmid DNA is a cGAS ligand, we analyzed bound pcDNA3, the plasmid backbone for the expression vectors, by qPCR at 3 different locations on the plasmid. Importantly the FLAG immunoprecipitation enriched cGAS at similar efficiency with and without SRC coexpression by Western blot (Figure 5B). immunoprecipitation qPCR percent input analysis revealed that FLAG-cGAS expression increased DNA binding as compared with mock-treated cells (Figure 5C). Intriguingly, SRC coexpression significantly reduced cGAS DNA binding at 3 locations on the plasmid as compared with cGAS expression alone. cGAS binding is unique to the transfected plasmids, as host *GAPDH* is not bound to cGAS under any condition. We further determined that cGAS DNA binding was reduced in an SRC kinase–dependent manner by performing a FLAG-cGAS immunoprecipitation qPCR with the K298M KD mutant, and we observed a rescue in DNA binding similar to EV levels (Figure 5D).

Lastly, to further examine SRC restriction of cGAS DNA binding, we isolated transfected cGAS ligands during SRC overexpression and analyzed bound cGAS. HEK293T cells expressing FLAG-cGAS with or without SRC-HA were transfected with biotinylated ISD45 and subjected to a streptavidin (strep) immunoprecipitation followed by Western blot analysis. Importantly, the strep immunoprecipitation of biotinylated ISD45 enriched for cGAS over the nonmodified ISD45, while SRC reduced cGAS binding in a kinase-dependent manner (Figure 5, E and F). Overall, these results demonstrate that SRC kinase activity limited cGAS DNA binding to restrict cGAS function.

### cGAS is phosphorylated by SRC.

**Figure 6:**
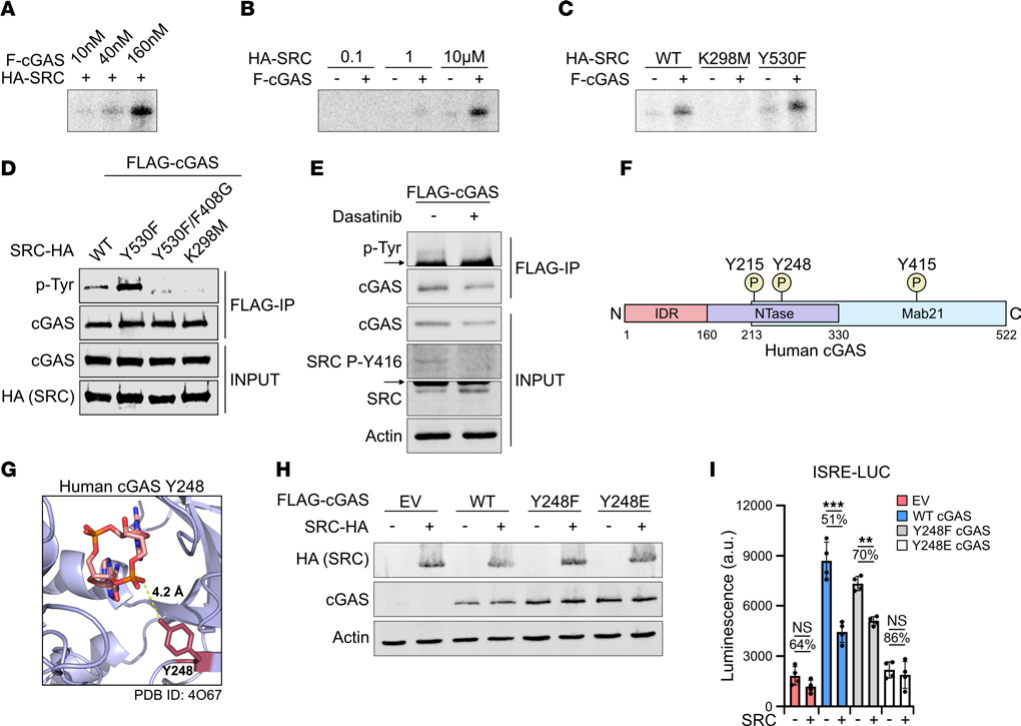
cGAS is phosphorylated by SRC, and cGAS Y248 is partially responsible for SRC-mediated inhibition. (**A**) In vitro kinase assay of 10 μM HA-SRC with various concentrations of FLAG-cGAS. Phosphorylation was analyzed by phosphor imaging. (**B**) In vitro kinase assay similar to **A** of 160 nM FLAG-cGAS with various concentrations of HA-SRC. (**C**) In vitro kinase assay similar to **A** of 160 nM FLAG-cGAS with 10 μM of WT and mutant HA-SRC. (**D**) Western blot of HEK293T cells transfected with FLAG-cGAS pcDNA3 and various SRC-HA pcDNA3 mutants for 24 hours, followed by FLAG IP and analysis of cGAS tyrosine phosphorylation. (**E**) Western blot similar to **D** with HEK293T cells treated with 30 nM dasatinib for 48 hours, followed by FLAG IP. Arrows depict nonspecific bands. (**F**) Schematic of human cGAS domains and observed tyrosine (Y) phosphorylation in PhosphoSitePlus. (**G**) Human cGAS structure (PDB ID: 4O67) with 2’3’ cGAMP in active site. Y248 is highlighted in red. (**H**) Western blot of HEK293T cells transfected with EV, WT, or Y248E/F FLAG-cGAS pcDNA3 mutants with or without SRC-HA pcDNA3 for 24 hours. (**I**) Coculture of L929-ISRE-LUC cells with cells in **G** (*n* = 4). Actin was used as a Western blot loading control. Statistical significance test: 1-way ANOVA with Tukey test (**I**). Representative assays shown. *n* = 3 (**I**).

Given that SRC directly interacts with cGAS, we sought to determine whether SRC phosphorylates cGAS. In vitro kinase assays were performed on purified FLAG-cGAS by HA-SRC, and phosphorylation was analyzed by phosphor imaging. FLAG-cGAS and HA-SRC are expressed at similar molecular weights; therefore, distinguishing between their phosphorylation status is difficult. Therefore, we varied the concentration of cGAS and SRC added to the reaction. Comparable concentrations of SRC incubated with increasing amounts of cGAS led to enhanced phosphorylation signal, presumably on cGAS (Figure 6A). Furthermore, the phosphorylation signal intensified as increasing amounts of SRC were added to the reaction (Figure 6B). Finally, WT and Y530F SRC phosphorylated cGAS while K298M did not, demonstrating that SRC phosphorylates cGAS in vitro (Figure 6C). To determine whether SRC also phosphorylates cGAS in cells, we transfected HEK293T cells with FLAG-cGAS and the various SRC-HA mutants, followed by a FLAG immunoprecipitation and analysis of tyrosine phosphorylation by Western blotting. Interestingly, WT SRC enhanced cGAS tyrosine phosphorylation, while the constitutively active Y530F SRC mutant further increased phosphorylation (Figure 6D). Importantly both Y530F/F408G and K298M KD mutants did not impact cGAS phosphorylation, suggesting that SRC kinase activity is responsible for tyrosine phosphorylation. We further determined that endogenous SRC phosphorylates cGAS in cells and that inhibiting SRC with dasatinib reduced cGAS tyrosine phosphorylation (Figure 6E).

To determine which cGAS tyrosine residues are phosphorylated by SRC and their impact on cGAS activity, we first analyzed known and observed modified sites using the PhosphoSitePlus database (Figure 6F) (54). cGAS Y215 is in the DNA binding domain, and phosphorylation promotes cytoplasmic localization to reduce a DNA damage–induced immune response as well as limits DNA binding (55, 56). cGAS Y248 and Y415 phosphorylation have only been detected using proteomic discovery mass spectrometry and have not been verified. Interestingly Y248 is in the cGAS enzymatic pocket and in close proximity to synthesized 2’3’ cGAMP (Figure 6G) (57). Since cGAS Y215, Y248, and Y415 are phosphorylated residues; Y215 and Y248 are in unique cGAS domains; and SRC interacts with the cGAS Mab21 domain, which contains the 3 tyrosine residues, we sought to determine what impact phosphorylation at these sites would have on SRC restriction of cGAS signaling. Tyrosine residues were mutated to phenylalanine to prevent phosphorylation or glutamic acid, which functions as a phosphomimetic. To determine what affect the cGAS tyrosine mutants have on SRC inhibition, HEK293T cells were transfected with WT or tyrosine mutant FLAG-cGAS with or without SRC-HA, followed by coculturing with L929-ISRE-LUC cells. Interestingly only Y248 mutants were expressed at similar levels with or without SRC, potentially due to reduced Y215 and Y415 mutant stability (Figure 6H). Therefore, we only investigated Y248, since reduced cGAS expression with SRC would not permit accurate analysis. While SRC again reduced WT cGAS signaling by ~49%, Y248F was only inhibited ~30%, suggesting that blocking Y248 phosphorylation restricts SRC inhibition (Figure 6I). Furthermore, Y248E was not active under any conditions. Collectively, these results demonstrate that cGAS was phosphorylated by SRC and that eliminating phosphorylation of cGAS Y248 partially blocked SRC inhibition of cGAS signaling.

In summary, SRC phosphorylates cGAS, which inhibits cGAS DNA binding, 2’3’ cGAMP production, and an innate immune response, while high SRC expression correlates with reduced immune gene expression and immune cell abundance in primary and metastatic tumors.

## Discussion

The cGAS-cGAMP-STING signaling pathway has garnered significant interest due to its pivotal roles during viral infection, in autoimmunity, and in the induction of antitumor immunity. Indeed, a hallmark of highly metastatic cancers is the presence of chromosomal instability, which results in the leakage of cytosolic dsDNA species through the formation of micronuclei (58). As such, tumor cells must employ a variety of strategies to dampen intrinsic cGAS-STING signaling in order to evade protective antitumor immune surveillance. Our results reveal a potentially novel mechanism of tumor immune evasion through direct phosphorylation of cGAS by the prototypical oncoprotein SRC. Moreover, using cancer cell lines and coculture models, we identified SRC inhibition as a promising strategy of augmenting cancer cell–intrinsic, as well as paracrine, innate immune activation in the context of cancer immunotherapy. By leveraging publicly available cancer genomics data, we found that enhanced tumor SRC expression was inversely correlated with type I IFN gene expression and immune cell infiltration. Intriguingly, our findings demonstrate that *SRC* levels did not correlate with reduced survival in bladder cancer. One potential reason for this result is the stringent expression cutoff that we employed in our analysis. Importantly, however, we found that expression of the oncogene *ERBB2* also did not correlate with bladder cancer survival, even though it negatively regulated cGAS signaling, implying that expression of immune-regulating oncogenes is not indicative of bladder cancer patient survival (33). Collectively, these data open up therapeutic avenues that could be leveraged to improve the immunogenicity of advanced and metastatic malignancies through the restoration of cancer cell–intrinsic cGAS signaling.

SRC — the first and most extensively studied oncogene — is the founding member of a 9-gene family of nonreceptor tyrosine kinases also known as SFKs, which are involved in the regulation of a variety of signaling processes. In the context of tumorigenesis, SRC has been implicated in mitogenic signaling, cell cycle progression, survival, angiogenesis, invasion, and metastasis as well as resistance to chemotherapeutics. Indeed, aberrant SRC activity has been identified in a variety of human malignancies, including melanoma, non–small cell lung cancer, urothelial malignancies, and breast cancers, among many others. There is an urgent need for a more detailed understanding of the cellular targets and processes modulated by oncogenic SRC signaling.

In recent years, strategies of augmenting STING signaling have been the subject of intense investigation for eliciting antitumor immune responses, especially for immunologically “cold” tumors through the activation of tumor-resident myeloid cells and vasculature (26, 28, 59, 60). The clinical importance of STING-mediated antitumor immunity is perhaps best exemplified by nonmuscle invasive bladder cancer where intravesical immunotherapy with the c-di-AMP–producing bacteria, bacillus Calmette-Guérin (BCG), is a mainstay of treatment (61, 62). In preclinical models of urothelial cancer, BCG strains engineered to overproduce c-di-AMP have yielded increased trained immunity, improved tumor clearance, and prolonged survival (63). Fascinatingly, our findings demonstrate that SRC negatively correlates with an immune response in primary bladder cancer and metastatic advanced urothelial carcinoma, suggesting that incorporating an SRC inhibitor with BCG treatment may enhance antitumor immunity. Additionally, STING agonists — including the synthetic, nonhydrolyzable cyclic dinucleotide analog, ADU-S100 — are currently under investigation for a variety of solid organ and hematologic malignancies either as monotherapy or in combination with immune checkpoint therapies. On the other hand, indiscriminate STING hyperactivation in noncancer cells may yield undesired off-target effects, including vasculitides, which may limit the patient tolerability of these agents. Furthermore, recent findings demonstrate that systemic delivery of cyclic dinucleotides, including 2’3’ cGAMP, repress antitumor immunity due to IL-35^+^ regulatory B cell expansion that restricts NK cell-mediated tumor killing (64). Our findings suggest that SRC inhibition could be leveraged to induce endogenous production of 2’3’ cGAMP in the tumor microenvironment (TME), which would eliminate the need for systemic treatment while still eliciting antitumor immunity.

Identification of cancer cell–specific strategies of cGAS-STING inhibition could, therefore, yield new therapeutic strategies for overcoming tumor-immune evasion and resistance to T cell–mediated immunotherapies. Indeed, repression of cGAS and STING expression through promoter hypermethylation has been observed across a variety of tumor types. To that end, pharmacological inhibition of DNA methylation has been proposed as one such strategy of restoring tumor cell intrinsic cGAS-STING signaling and downstream cytotoxic T cell infiltration and killing (65). Similarly, upregulation of the cGAMP phosphodiesterase, ectonucleotide pyrophosphatase/phosphodiesterase 1 (ENPP1), has been proposed as a mechanism by which highly metastatic tumors evade immune surveillance programs while simultaneously inducing an immunosuppressive profile (58). Thus, ENPP1 inhibitors are currently under investigation in preclinical models as well as clinical trials for patients with advanced, unresectable, or metastatic tumors.

Conversely, mechanisms by which tumor cells directly modulate DNA sensing and activity of cGAS remain poorly understood. The discovery of SRC-mediated phosphorylation and inhibition of cGAS highlights an important strategy of tumor immune evasion and may provide mechanistic insights into how SRC inhibition improves T cell infiltration and immune responses in immunogenic tumors (66). Moreover, our data suggest that SRC inhibitors could be employed as adjuvants or as a rescue strategy to improve tumor immunogenicity during immune checkpoint blockade and/or radiation therapy (67). Intriguingly, dasatinib treatment also induces an immunosuppressive response by directly inhibiting NK and T cell function in murine models, demonstrating a potential clinical limitation and suggesting that tumor-localized SRC inhibition may be necessary to activate a tumor-derived immune response and avoid off-target systemic immunosuppression (68). Although not tested here, cGAS tyrosine phosphorylation by other oncogenic SFKs may be a common strategy employed by cancers to promote an immunosuppressive phenotype. Indeed, inhibition of the myeloid-specific SFK hematopoietic cell kinase (HCK) was recently shown to license T cell–mediated immunotherapies in refractory tumors through remodeling of the immunosuppressive TME (66).

More generally, it is intriguing to speculate that other growth factor receptor tyrosine kinases and oncogenic signaling pathways may also exhibit inhibitory effects on innate immune sensing pathways in order to promote a noninflamed TME. In support of this, oncogenic MYC signaling has been shown to induce epigenetic silencing of cGAS-STING signaling in triple-negative breast cancer (69). Similarly, the ERBB2 pathway disrupts STING signaling through AKT-mediated phosphorylation and dysregulation of TBK1, while combination targeted therapy with the tyrosine kinase inhibitor Osimertinib and monoclonal antibodies targeting ERB-B2 receptor tyrosine kinase 3 (HER3) was recently shown to induce enhanced tumor cell cGAMP production and paracrine STING activation in tumor-associated macrophages to facilitate tumor clearance (33, 70). Thus, a more detailed understanding of cancer subtype–specific strategies of immune evasion will enable the generation of rationally designed combination therapies aimed at restoring tumor immunogenicity.

## Methods

### Cells and viruses

A549 (ATCC), HEK293T (ATCC), HT29 (ATCC), 1.1B4 (ATCC), and L929-ISRE-LUC (40) cells were maintained in DMEM (Invitrogen) supplemented with 10% FBS (Genesee). THP-1 (ATCC), B16-BL6 (26), and ASPC1 (ATCC) cells were grown in RPMI 1640 (Invitrogen) medium supplemented with 10% FBS (Genesee). All cells were maintained with 100 U of penicillin/mL and 100 μg of streptomycin/mL (Invitrogen) at 37°C under 5% CO_2_. THP-1 cells were differentiated with 150 nM PMA for 24 hours, followed by removing drug and resting cells in fresh media for 24 hours.

### SRC inhibitor treatment

Cells were treated with 30 nM dasatinib (MilliporeSigma), 80 nM saracatinib (Selleck), or vehicle for 48 hours. Each day, media were replaced with fresh drug. SRC inhibition by dasatinib was verified in A549 and HEK293T cells by analyzing SRC Y416 phosphorylation by Western blot.

### Nucleic acid ligand and plasmid transfection

Cells were transfected at 70%–80% confluence with 10 μg/mL HT-DNA (MilliporeSigma), 3 μg/mL ISD45 (Integrated DNA Technologies), 1 μg/mL poly(I:C) (Invivogen), or 2 μg plasmid DNA using Lipofectamine 2000 (Invitrogen). HT-DNA and ISD45 samples were collected 6 hours after transfection, while poly(I:C) samples were collected 16 hours after transfection. Cells transfected with plasmid DNA had fresh media added 6 hours after transfection.

HEK293T cells were transfected at 60%–80% confluence with 1 μg (6-well) or 6 μg (10 cm dish) plasmid DNA using 4 μg (6-well) or 24 μg (10 cm dish) PEI (Polysciences). Samples were collected 24 hours after transfection.

### ISRE-LUC assay

HEK293T and A549 cells transfected with HT-DNA, ISD45, or vehicle for 6 hours were cocultured with L929-ISRE-LUC cells for 24 hours at 37°C unless otherwise stated. Cells were lysed in LUC lysis buffer (50 mM Tris [pH 7.5], 150 mM NaCl, 1 mM EDTA, 1% NP-40), and cell debris was pelleted by centrifugation at 21,000*g* for 10 seconds. Supernatant was transferred to a white-walled 96-well plate (Life Science Products) and combined with LUC buffer (20 mM Tricine from MilliporeSigma, 2.67 mM MgSO from Thermo Fisher Scientific, 4.7 H_2_O, 0.1 mM EDTA from Thermo Fisher Scientific, 33.3 mM DTT from Thermo Fisher Scientific, 530 μM ATP from Thermo Fisher Scientific, 270 μM acetyl CoA lithium salt from MilliporeSigma, 470 μM luciferin from Biosynth, 5 mM NaOH from Thermo Fisher Scientific, 265 μM magnesium carbonate hydroxide from MilliporeSigma). LUC activity was monitored by a BioTek synergy HTX plate reader.

### Cloning and cell line generation

Human *SRC* gene was amplified by PCR from pJP1520-SRC retroviral expression vector (DNASU) and restriction digest cloned into pcDNA3 vector to generate SRC-HA-pcDNA3. SRC-HA-pcDNA3 underwent site-directed mutagenesis (SDM) to generate constitutively active (Y530F) and KD (K298M and F408G/Y530F) mutants. SRC truncations were cloned following amplification from SRC-HA-pcDNA3 and restriction digest cloned into pcDNA3.

WT and mutant SRC were further amplified from SRC-HA-pcDNA3 and restriction digest cloned into pSB-tet-RB (44). A549 and THP-1 cells were transfected with WT and mutant HA-SRC pSB-tet-RB and SB100X (Addgene) followed by blasticidin (Thermo Fisher Scientific) selection. His-HA-SRC pSB-tet-RB was generated by SDM and transfected into HEK293T with SB100X followed by blasticidin selection.

Human *cGAS* was amplified by PCR from cGAS pcDNA3 (provided by Genhong Cheng, UCLA, Los Angeles, California, USA) and restriction digest cloned into pcDNA3 vector to generate FLAG-cGAS-pcDNA3. cGAS truncations were cloned following amplification from FLAG-cGAS pcDNA3 and restriction digest cloned into pcDNA3. FLAG-cGAS-pcDNA3 underwent SDM to generate Y248E/F mutants. pSUMO2 human cGAS-FL (provided by Philip Kranzusch, Harvard Medical School, Boston, Massachusetts, USA) underwent SDM to insert a FLAG tag and generate His-FLAG-cGAS pSUMO2.

### Western blotting

Cell lysates were prepared with LUC lysis buffer and quantified by BCA assay (Thermo Fisher Scientific). Equivalent amounts of each sample were resolved by SDS-PAGE, electrotransferred to nitrocellulose membrane, and blotted for indicated proteins. Primary antibodies (listed in Supplemental Table 4) were followed by Alexa Fluor 680– or Alexa Fluor 800–conjugated anti-rabbit (Li-Cor 926-32211 and 926-68071) and anti-mouse (Li-Cor 926-68070 and 926-32210) secondary antibodies and visualized by Li-Cor Odyssey.

### Nucleic acid isolation and measurement

For analysis of gene expression by qPCR, total RNA was isolated with Takara NucleoSpin RNA plus kit in accordance with manufacturer’s instructions. cDNA was synthesized from RNA with random primer (Invitrogen) and Maxima H minus RT (Thermo Fisher Scientific). qPCR was performed using the PowerUp SYBR Green qPCR kit (Thermo Fisher Scientific) with appropriate primers (Supplemental Table 4).

### CRISPR-Cas9 and pLKO-tet-On-shRNA cloning, lentiviral production, and infection

sgRNAs (Supplemental Table 4) were selected by inputting *cGAS* gene sequence into the Broad Institute GPP sgRNA CRISPR KO Designer. Two high-score sgRNAs were selected. sgRNA oligos were cloned into lentiCRISPR V2 (Addgene) lentiviral plasmid according to depositor instructions.

shRNAs (Supplemental Table 4) were selected using the Broad Institute Genetic Perturbation Platform. Two high-score shRNAs were selected for SRC. shRNA oligos were cloned into pLKO-Tet-On-shRNA (Addgene) lentiviral plasmid according to depositor instructions.

Lentivirus was prepared in HEK293T cells. Cells were transfected at 50%–60% confluence with lentiCRISPR V2, psPAX2 (lentiviral packaging), and pVSV-G (lentiviral envelope) (Addgene). Seventy-two hours after transfection, supernatant was collected, mixed with 8 μg/mL of polybrene and 0.5% PEG, and added to HEK293T or A549 cells that were spinfected at 280*g* for 1 hour at room temperature. Cells were selected for 2 weeks in media containing 2 μg/mL puromycin (Thermo Fisher Scientific).

### KO generation

Single-cell clones were grown out in 96-well plates. KO was verified by transfecting poly(I:C) followed by analysis of cGAS levels by Western blot. CRISPR-Cas9–induced mutations were identified by isolation of genomic DNA (Promega), PCR of genomic region where the guide RNA targets, TOPO cloning, and Sanger sequencing of PCR product.

### NF-κB activity LUC assay

HEK293T were transfected with pNiFty-LUC (Invivogen), eGFP pcDNA3 (Addgene), FLAG-cGAS pcDNA3, and various SRC-HA pcDNA3 mutants for 24 hours. LUC activity was analyzed similarly as ISRE-LUC assay. GFP levels were analyzed as a transfection control.

### Immunoprecipitations and co-immunoprecipitation

HEK293T cells were transfected in a 10 cm dish with equal concentrations of EV (pcDNA3), FLAG-cGAS pcDNA3, or SRC-HA pcDNA3. Twenty-four hours after transfection, cells were collected and lysed in co-immunoprecipitation lysis buffer (50 mM Tris [pH 7.5], 150 mM NaCl, 10% glycerol, 0.5% NP-40), and cell debris was pelleted by centrifugation at 21,000*g* for 10 minutes. Cell extract was incubated with FLAG M2 magnetic beads (MilliporeSigma) or HA magnetic beads (Thermo Fisher Scientific) for 1 hour at 4°C under rotation. Beads were washed 3 times for 10 min with co-immunoprecipitation lysis buffer. Resin was eluted with 1× FLAG peptide (MilliporeSigma) or HA peptide (Thermo Fisher Scientific) in co-immunoprecipitation buffer for 1 hour at 4°C. Co-immunoprecipitated proteins were analyzed by Western blot.

THP-1 WT HA-SRC pSB-tet-RB cells were treated with 1 μg/mL DOX for 48 hours and transfected with poly(I:C) for 16 hours. Co-immunoprecipitation was executed similarly to HEK293T co-immunoprecipitation, and resin was eluted with HA peptide.

In total, 4 μg of purified FLAG-cGAS and HA-SRC were incubated in in vitro co-immunoprecipitation buffer (20 mM Tris [pH 7.5] from MilliporeSigma, 150 mM NaCl from Thermo Fisher Scientific, 3 mM EDTA from Thermo Fisher Scientific, 3 mM EGTA from Thermo Fisher Scientific, 0.5% NP-40 from ICN Biomedical, protease inhibitor cocktail from Thermo Fisher Scientific) for 1 hour. Proteins were added to FLAG M2 magnetic beads and incubated overnight at 4°C under rotation. Beads were washed 3 times for 10 minutes with in vitro co-immunoprecipitation lysis buffer. Resin was eluted with 1× FLAG peptide.

#### SRC overexpression.

HEK293T cells were transfected in a 60 mm dish with equal concentrations of FLAG-cGAS pcDNA3 and SRC-HA pcDNA3 mutants.

#### SRC inhibition.

HEK293T cells were treated with 30 nM dasatinib for 24 hours prior to FLAG-cGAS transfection. Cells were lysed 24 hours after transfection as described above, followed by FLAG immunoprecipitation and elution. cGAS tyrosine phosphorylation status was analyzed by Western blot.

### Purification of FLAG-cGAS, HA-SRC mutants, BioSTING

Human FLAG-cGAS was purified from LOBSTR *E*. *coli* expression competent cells as previously described (71). Purity was analyzed by Coomassie Brilliant Blue staining. Concentrated aliquots were flash frozen and stored at −80°C. Activity was analyzed by in vitro FRET assay.

Human WT and mutant His-HA-SRC pSB-tet-RB HEK293T cells were treated with 2 μg/mL DOX for 24 hours. Cells were treated with 1 μM dasatinib 1 hour prior to lysis. Cells were lysed in SRC lysis buffer (20 mM Tris [pH 7.5], 1% Triton X-100, 10% glycerol, 400 mM NaCl, 1 mM EDTA, 20 mM imidazole, 1 mM DTT, 1 mM AEBSF, 10 μM sodium vanadate), and cell debris was pelleted by centrifugation at 21,000*g* for 10 minutes. Supernatant was bound to His-Pur Ni-NTA Resin (Thermo Fisher Scientific). Resin was washed with 120 bed volumes of lysis buffer and eluted in SRC elution buffer (20 mM Tris [pH 7.5], 1% Triton X-100, 10% glycerol, 400 mM NaCl, 1 mM EDTA, 300 mM imidazole, 1 mM DTT, 1 mM AEBSF, 10 μM sodium vanadate). Eluted protein was concentrated and subjected to buffer exchange into SRC storage buffer (50 mM Tris [pH 7.5], 150 mM NaCl, 1 mM EDTA, 1 mM DTT). Purity was analyzed by Coomassie staining. Glycerol was added to a final 50% volume, and aliquots were stored at −20°C. Activity was analyzed by autophosphorylation via in vitro kinase assays.

BioSTING was purified from LOBSTR *E*. *coli* expression competent cells as previously described (52). Purity was analyzed by Coomassie staining. Concentrated aliquots were flash frozen and stored at −80°C. Activity was analyzed by in vitro FRET assay.

### EIA and ELISA

#### 2’3’ cGAMP EIA.

HEK293T cells were transfected with FLAG-cGAS pcDNA3 and indicated SRC-HA pcDNA3 mutants for 24 hours. Cells were washed with cold PBS, and lysates were prepared in accordance with the manufacturer’s instructions (Arbor Assays).

#### IFNB and TNFA ELISA.

THP-1 WT HA-SRC pSB-tet-RB cells were treated with 1 μg/mL DOX for 48 hours. Cells were transfected with 3 μg/mL ISD45 or 10 μg/mL HT-DNA for 24 hours, followed by supernatant collection and analysis of IFNB and TNFA protein concentrations in accordance with the manufacturer’s instructions (Proteintech).

### In vitro kinase assay

WT and mutant HA-SRC was incubated with FLAG-cGAS in kinase buffer (5 mM MOPS [pH 7.2], 2.5 mM β-glycerol-phosphate, 4 mM MgCl_2_, 2.5 mM MnCl_2_, 1 mM EGTA, 0.4 mM EDTA, 10 mM DTT, 50 μM ATP, 4 μCi γ-32P ATP) at 30°C for 30 minutes. Nonradioactive assays were conducted with 250 μM ATP. Endpoint assay reactions were stopped by adding 4X SDS-PAGE loading dye at 95°C for 5 minutes, followed by SDS-PAGE analysis. Radioactive SDS-PAGE gels were imaged on Azure Sapphire imager.

### FRET assays

#### Cellular.

HEK293T cells were transfected with FLAG-cGAS pcDNA3 and SRC-HA pcDNA3 mutants indicated in figure for 24 hours. Cells were lysed in LUC lysis buffer at 4°C for 20 minutes, cell debris was pelleted by centrifugation at 21,000*g* for 10 minutes, and supernatants were incubated with 1 μM of purified BioSTING in BioSTING activity buffer (50 mM Tris [pH 7.5], 35 mM KCl, 5 mM Mg[OAc]_2_). Enzyme assays proceeded for 2 hours at 37°C, and FRET fluorescence was monitored using FRET-capable plate reader (CLARIOstar) with the following parameters: 458 nm excitation, 490 nm, and 600 nm emission.

#### In vitro.

30 nM FLAG-cGAS was phosphorylated by WT HA-SRC using above in vitro kinase assay. Reaction was combined with 1 μM of purified BioSTING, 0.5 ng ISD45, 25 μM ATP, 25 μM GTP in BioSTING activity buffer.

### FLAG-cGAS ChIP

HEK293T cells were transfected with FLAG-cGAS pcDNA3, EV pcDNA3, and SRC-HA pcDNA3 for 24 hours. Cells were crosslinked in 1% formaldehyde (VWR) for 10 minutes, quenched with glycine, and snap-frozen. Cell pellets were lysed in SDS lysis buffer (0.8% SDS, 10 mM EDTA, 50 mM Tris [pH 8.1], protease inhibitor cocktail), then sonicated, and cell debris was pelleted by centrifugation at 21,000*g* for 10 minutes. Supernatant was diluted 3-fold in dilution buffer (0.003% SDS, 0.4 mM EDTA, 5.6 mM Tris [pH 8.1], 3.3% Triton X-100, 501 mM NaCl, protease inhibitor cocktail), and incubated with FLAG M2 magnetic beads overnight at 4°C under rotation. Beads were washed 2 times each in following buffers: low salt (0.1% SDS, 2 mM EDTA, 20 mM Tris [pH 8.1], 1% Triton X-100, 150 mM NaCl), high salt (0.1% SDS, 2 mM EDTA, 20 mM Tris [pH 8.1], 1% Triton X-100, 500 mM NaCl), LiCl (0.25 M LiCl, 1 mM EDTA, 10 mM Tris [pH 8.1], 1% NP-40, 1% sodium deoxycholic acid), and Tris-EDTA (1 mM EDTA, 10 mM Tris [pH 8.1]) buffer. Input and beads were resuspended in elution buffer (1% SDS, 150 mM NaCl) supplemented with 150 μg/mL 1× FLAG peptide, and crosslinks were reversed by incubating at 65°C overnight. Following bead removal by magnet, DNA was isolated from 90% of the supernatant by adding 50 μg/mL proteinase K (Thermo Fisher Scientific) and RNase A (NEB) at 65°C for 2 hours followed by PCR clean-up. Bound pcDNA3 was analyzed by qPCR. The remaining 10% was treated with DNase (Invitrogen) and RNase A at 37°C and analyzed by Western blot.

### Biotin-ISD45 annealing and immunoprecipitation

Biotinylated forward and nonmodified reverse ISD45 oligos (Integrated DNA Technologies) were resuspended at 1 mg/mL in annealing buffer (5 mM Tris [pH 7.5], 25 mM NaCl) and annealed by incubating oligos together at 95°C for 5 minutes before being cooled to 25°C at 0.1°C/s.

HEK293T cells were transfected with FLAG-cGAS pcDNA3, EV pcDNA3, and SRC-HA pcDNA3 for 24 hours. In total, 4 μg/mL annealed ISD45 was transfected into cells using PEI. Two hours later, cells were washed in PBS and lysed in biotin-ISD45 lysis buffer (20 mM Tris [pH 7.5], 100 mM NaCl, 3 mM EDTA, 3 mM EGTA, 0.5% NP-40, 0.1% BSA, protease inhibitor cocktail), followed by centrifugation at 21,000*g* for 10 minutes. Cell lysates were incubated with streptavidin magnetic beads (NEB) for 1 hour at 4°C under rotation. Beads were washed 5 times in lysis buffer, and protein was eluted off resin using 1× SDS-PAGE loading dye and heating at 95°C for 7 minutes.

### Genome annotations and gene expression estimation

Human genome annotation was created for UCSC hg19 (GRCh37) assembly by merging UCSC knownGene, Ensembl 71, and MISO v2.0 annotations (72–74). RNA-Seq reads were mapped to transcriptome using RSEM v1.2.4, invoking Bowtie alignment with the option “-v 2” (75, 76). Unaligned reads were mapped to genome with TopHat v2.0.8b and to an annotation generated from all possible combinations of annotated 5′ and 3′ splice sites, per gene (77). The gene expression estimates produced (transcripts per million [TPM]) were normalized using trimmed mean of M values method (78).

### Differential gene expression, gene ontology enrichment, and gene signature analyses

Gene expression values per sample group were compared using 2-sided Mann-Whitney *U* test. Differentially expressed genes were defined as those with an absolute log_2_(fold-change) ≥ log_2_(1.5) and *P* < 0.05. Using differentially expressed genes as input and a FDR threshold of 0.01, enriched gene ontology (GO) terms associated with up- or downregulated genes were enumerated via GOseq (79). Significant GO terms were defined as terms under the “Biological Process” ontology harboring Benjamini-Hochberg FDR-adjusted *P* < 0.05. In the analyses that utilize gene signatures, a signature score was calculated for a given gene set: mean of the normalized expression values across the genes of set. In correlation analyses comparing IFN-α/β pathway activity with gene expression, multiple-hypothesis testing correction was performed via Benjamini-Yekutieli method for FDR control (80). Visualizations were created in the R programming environment using dplyr and ggplot2 packages.

### Immune cell abundance estimation

Abundance of immune cells in TME was estimated from bulk RNA-Seq data of primary bladder cancer samples from TCGA using CIBERSORT, implemented through the immunedeconv package (38, 81). LM22 leukocyte gene signature matrix, obtained from original study, and a matrix of gene expression values in TPM units were used as input (38). Immune cell fractions between sample groups were compared using 2-sided Mann-Whitney *U* test. Visualizations were created in the R programming environment using dplyr and ggplot2 packages.

### Survival analysis

Kaplan-Meier estimation of survival and *P* value estimation from the log-rank test were performed using the survival package (82). Visualizations were created in the R programming environment using the dplyr and ggplot2 packages.

### Code availability

RNA-Seq reads from TCGA, the IMVigor210 clinical trial, and the Illumina Body Map 2 Project were downloaded from the Cancer Genomics Hub, the European Genome-Phenome Archive (accession number: EGAD00001003977), and the European Nucleotide Archive (accession no. ERP000546), respectively (39). This paper does not report original code.

### Statistics

Statistical parameters are reported in the figures and corresponding figure legends. Student’s *t* test was used to compare means between 2 groups, and 1-way ANOVA with Tukey test was used to compare means among 3 or more groups. All Student’s *t* tests are 2-tailed tests. *P ≤ 0.05; **P ≤ 0.005; ***P ≤ 0.0005. Results were expressed as mean ± SD. Value of *n* is depicted in each figure legend. LUC assays were performed using the BioTek synergy HTX plate reader. FRET assays were performed using the CLARIOstar plate reader. qPCR was performed using the CFX Connect Real-Time system (Bio-Rad). Immunoblots were developed using the Odyssey. Phosphor imaging was performed on the Azure Sapphire.

### Data availability

Any additional information required to reanalyze the data reported in this paper is available from the lead contact upon request.

## Author contributions

WD, SAZ, and JJW conceived and designed the experiments. WD, SAZ, and CJH performed the experiments. WD and SAZ analyzed the data. JMBP and RKB analyzed bioinformatics. WD, SAZ, and JJW wrote the paper.

## Supplementary Material

Supplemental data

Supplemental table 1

Supplemental table 2

Supplemental table 3

Supplemental table 4

Supporting data values

## Version 1 05/11/2023

In-Press Preview

## Version 2 06/22/2023

Electronic publication

## References

[1] Tang D (2012). PAMPs and DAMPs: signal 0s that spur autophagy and immunity. Immunol Rev.

[2] Sun L (2013). Cyclic GMP-AMP synthase is a cytosolic DNA sensor that activates the type I interferon pathway. Science.

[3] Wu J (2013). Cyclic GMP-AMP is an endogenous second messenger in innate immune signaling by cytosolic DNA. Science.

[4] Takaoka A (2007). DAI (DLM-1/ZBP1) is a cytosolic DNA sensor and an activator of innate immune response. Nature.

[5] Bürckstümmer T (2009). An orthogonal proteomic-genomic screen identifies AIM2 as a cytoplasmic DNA sensor for the inflammasome. Nat Immunol.

[6] Ferguson BJ (2012). DNA-PK is a DNA sensor for IRF-3-dependent innate immunity. Elife.

[7] Han F (2021). The cGAS-STING signaling pathway contributes to the inflammatory response and autophagy in Aspergillus fumigatus keratitis. Exp Eye Res.

[8] Hopfner K-P, Hornung V (2020). Molecular mechanisms and cellular functions of cGAS-STING signalling. Nat Rev Mol Cell Biol.

[9] Ablasser A (2013). cGAS produces a 2’-5’-linked cyclic dinucleotide second messenger that activates STING. Nature.

[10] Zhang X (2013). Cyclic GMP-AMP containing mixed phosphodiester linkages is an endogenous high-affinity ligand for STING. Mol Cell.

[11] Diner EJ (2013). The innate immune DNA sensor cGAS produces a noncanonical cyclic dinucleotide that activates human STING. Cell Rep.

[12] Gao P (2013). Cyclic [G(2’,5’)pA(3’,5’)p] is the metazoan second messenger produced by DNA-activated cyclic GMP-AMP synthase. Cell.

[13] Ishikawa H, Barber GN (2008). STING is an endoplasmic reticulum adaptor that facilitates innate immune signalling. Nature.

[14] Ablasser A (2014). TREX1 deficiency triggers cell-autonomous immunity in a cGAS-dependent manner. J Immunol.

[15] Ahn J (2014). Intrinsic self-DNA triggers inflammatory disease dependent on STING. J Immunol.

[16] Gray EE (2015). Cutting Edge: cGAS is required for lethal autoimmune disease in the Trex1-deficient mouse model of Aicardi-Goutières syndrome. J Immunol.

[17] Gao D (2015). Activation of cyclic GMP-AMP synthase by self-DNA causes autoimmune diseases. Proc Natl Acad Sci U S A.

[18] Mackenzie KJ (2016). Ribonuclease H2 mutations induce a cGAS/STING-dependent innate immune response. EMBO J.

[19] Liang Q (2014). Crosstalk between the cGAS DNA sensor and Beclin-1 autophagy protein shapes innate antimicrobial immune responses. Cell Host Microbe.

[20] Hu M-M (2016). Sumoylation promotes the stability of the DNA sensor cGAS and the adaptor STING to regulate the kinetics of response to DNA virus. Immunity.

[21] Seo GJ (2015). Akt kinase-mediated checkpoint of cGAS DNA sensing pathway. Cell Rep.

[22] Zhong L (2020). Phosphorylation of cGAS by CDK1 impairs self-DNA sensing in mitosis. Cell Discov.

[23] Li T (2021). Phosphorylation and chromatin tethering prevent cGAS activation during mitosis. Science.

[24] Kwon J, Bakhoum SF (2020). The cytosolic DNA-sensing cGAS-STING pathway in cancer. Cancer Discov.

[25] Yang H (2017). cGAS is essential for cellular senescence. Proc Natl Acad Sci U S A.

[26] Marcus A (2018). Tumor-derived cGAMP triggers a STING-mediated interferon response in non-tumor cells to activate the NK cell response. Immunity.

[27] Garland KM (2021). Pharmacological activation of cGAS for cancer immunotherapy. Front Immunol.

[28] Corrales L (2015). Direct activation of STING in the tumor microenvironment leads to potent and systemic tumor regression and immunity. Cell Rep.

[29] Wang H (2017). cGAS is essential for the antitumor effect of immune checkpoint blockade. Proc Natl Acad Sci U S A.

[30] Flood BA (2019). STING pathway agonism as a cancer therapeutic. Immunol Rev.

[31] Konno H (2018). Suppression of STING signaling through epigenetic silencing and missense mutation impedes DNA damage mediated cytokine production. Oncogene.

[32] Wu M-Z (2017). miR-25/93 mediates hypoxia-induced immunosuppression by repressing cGAS. Nat Cell Biol.

[33] Wu S (2019). HER2 recruits AKT1 to disrupt STING signalling and suppress antiviral defence and antitumour immunity. Nat Cell Biol.

[34] Ishizawar R, Parsons SJ (2004). c-Src and cooperating partners in human cancer. Cancer Cell.

[35] Irby RB, Yeatman TJ (2000). Role of Src expression and activation in human cancer. Oncogene.

[36] Belli S (2020). c-Src and EGFR inhibition in molecular cancer therapy: what else can we improve?. Cancers (Basel).

[37] Gentles AJ (2015). The prognostic landscape of genes and infiltrating immune cells across human cancers. Nat Med.

[38] Newman AM (2015). Robust enumeration of cell subsets from tissue expression profiles. Nat Methods.

[39] Mariathasan S (2018). TGFβ attenuates tumour response to PD-L1 blockade by contributing to exclusion of T cells. Nature.

[40] Woodward JJ (2010). c-di-AMP secreted by intracellular Listeria monocytogenes activates a host type I interferon response. Science.

[41] Ablasser A (2013). Cell intrinsic immunity spreads to bystander cells via the intercellular transfer of cGAMP. Nature.

[42] Eaglesham JB (2019). Viral and metazoan poxins are cGAMP-specific nucleases that restrict cGAS-STING signalling. Nature.

[43] Kmiecik TE (1988). Regulation by the autophosphorylation site in overexpressed pp60c-src. Mol Cell Biol.

[44] Kowarz E (2015). Optimized sleeping beauty transposons rapidly generate stable transgenic cell lines. Biotechnol J.

[45] Agius MP (2019). Selective proteolysis to study the global conformation and regulatory mechanisms of c-Src kinase. ACS Chem Biol.

[46] Cartwright CA (1987). Cell transformation by pp60c-src mutated in the carboxy-terminal regulatory domain. Cell.

[47] Kmiecik TE, Shalloway D (1987). Activation and suppression of pp60c-src transforming ability by mutation of its primary sites of tyrosine phosphorylation. Cell.

[48] Piwnica-Worms H (1987). Tyrosine phosphorylation regulates the biochemical and biological properties of pp60c-src. Cell.

[49] Snyder MA (1985). A mutation at the ATP-binding site of pp60v-src abolishes kinase activity, transformation, and tumorigenicity. Mol Cell Biol.

[50] Parsons SJ, Parsons JT (2004). Src family kinases, key regulators of signal transduction. Oncogene.

[51] Rehwinkel J, Gack MU (2020). RIG-I-like receptors: their regulation and roles in RNA sensing. Nat Rev Immunol.

[52] Pollock AJ (2020). A STING-based biosensor affords broad cyclic dinucleotide detection within single living eukaryotic cells. Nat Commun.

[53] Guey B (2020). BAF restricts cGAS on nuclear DNA to prevent innate immune activation. Science.

[54] Hornbeck PV (2015). PhosphoSitePlus, 2014: mutations, PTMs and recalibrations. Nucleic Acids Res.

[55] Liu H (2018). Nuclear cGAS suppresses DNA repair and promotes tumorigenesis. Nature.

[56] Jiang H (2019). Chromatin-bound cGAS is an inhibitor of DNA repair and hence accelerates genome destabilization and cell death. EMBO J.

[57] Zhang X (2014). The cytosolic DNA sensor cGAS forms an oligomeric complex with DNA and undergoes switch-like conformational changes in the activation loop. Cell Rep.

[58] Li J (2021). Metastasis and immune evasion from extracellular cGAMP hydrolysis. Cancer Discov.

[59] Mekers VE (2022). cGAS/cGAMP/STING signal propagation in the tumor microenvironment: key role for myeloid cells in antitumor immunity. Radiother Oncol.

[60] Campisi M (2020). Tumor-derived cGAMP regulates activation of the vasculature. Front Immunol.

[61] Huang K-C (2022). Pharmacologic activation of STING in the bladder induces potent antitumor immunity in non-muscle invasive murine bladder cancer. Mol Cancer Ther.

[62] Lombardo KA (2022). BCG invokes superior STING-mediated innate immune response over radiotherapy in a carcinogen murine model of urothelial cancer. J Pathol.

[63] Singh AK (2022). Re-engineered BCG overexpressing cyclic di-AMP augments trained immunity and exhibits improved efficacy against bladder cancer. Nat Commun.

[64] Li S (2022). STING-induced regulatory B cells compromise NK function in cancer immunity. Nature.

[65] Falahat R (2021). Epigenetic reprogramming of tumor cell-intrinsic STING function sculpts antigenicity and T cell recognition of melanoma. Proc Natl Acad Sci U S A.

[66] Poh AR (2022). Therapeutic inhibition of the SRC-kinase HCK facilitates T cell tumor infiltration and improves response to immunotherapy. Sci Adv.

[67] Redin E (2021). SRC family kinase (SFK) inhibitor dasatinib improves the antitumor activity of anti-PD-1 in NSCLC models by inhibiting Treg cell conversion and proliferation. J Immunother Cancer.

[68] Fraser CK (2009). Dasatinib inhibits recombinant viral antigen-specific murine CD4+ and CD8+ T-cell responses and NK-cell cytolytic activity in vitro and in vivo. Exp Hematol.

[69] Wu S-Y (2021). MYC suppresses STING-dependent innate immunity by transcriptionally upregulating DNMT1 in triple-negative breast cancer. J Immunother Cancer.

[70] Vicencio JM (2022). Osimertinib and anti-HER3 combination therapy engages immune dependent tumor toxicity via STING activation in trans. Cell Death Dis.

[71] Zhou W (2018). Structure of the human cGAS-DNA complex reveals enhanced control of immune surveillance. Cell.

[72] Katz Y (2010). Analysis and design of RNA sequencing experiments for identifying isoform regulation. Nat Methods.

[73] Flicek P (2013). Ensembl 2013. Nucleic Acids Res.

[74] Meyer LR (2013). The UCSC Genome Browser database: extensions and updates 2013. Nucleic Acids Res.

[75] Langmead B (2009). Ultrafast and memory-efficient alignment of short DNA sequences to the human genome. Genome Biol.

[76] Li B, Dewey CN (2011). RSEM: accurate transcript quantification from RNA-Seq data with or without a reference genome. BMC Bioinformatics.

[77] Trapnell C (2009). TopHat: discovering splice junctions with RNA-Seq. Bioinformatics.

[78] Robinson MD, Oshlack A (2010). A scaling normalization method for differential expression analysis of RNA-seq data. Genome Biol.

[79] Young MD (2010). Gene ontology analysis for RNA-seq: accounting for selection bias. Genome Biol.

[80] Benjamini Y, Yekutieli D (2001). The control of the false discovery rate in multiple testing under dependency. Ann Stat.

[81] Sturm G (2019). Comprehensive evaluation of transcriptome-based cell-type quantification methods for immuno-oncology. Bioinformatics.

[82] https://CRAN.R-project.org/package=survival.

