# Supplementary material for: The proto-oncogene SRC phosphorylates cGAS to inhibit an antitumor immune response
Source: JCI Insight. 2023 Jun 22;8(12):e167270. doi: 10.1172/jci.insight.167270 (PMC10371251; doi:10.1172/jci.insight.167270)
Supplement: Supplemental data [file jciinsight-8-167270-s186.pdf]

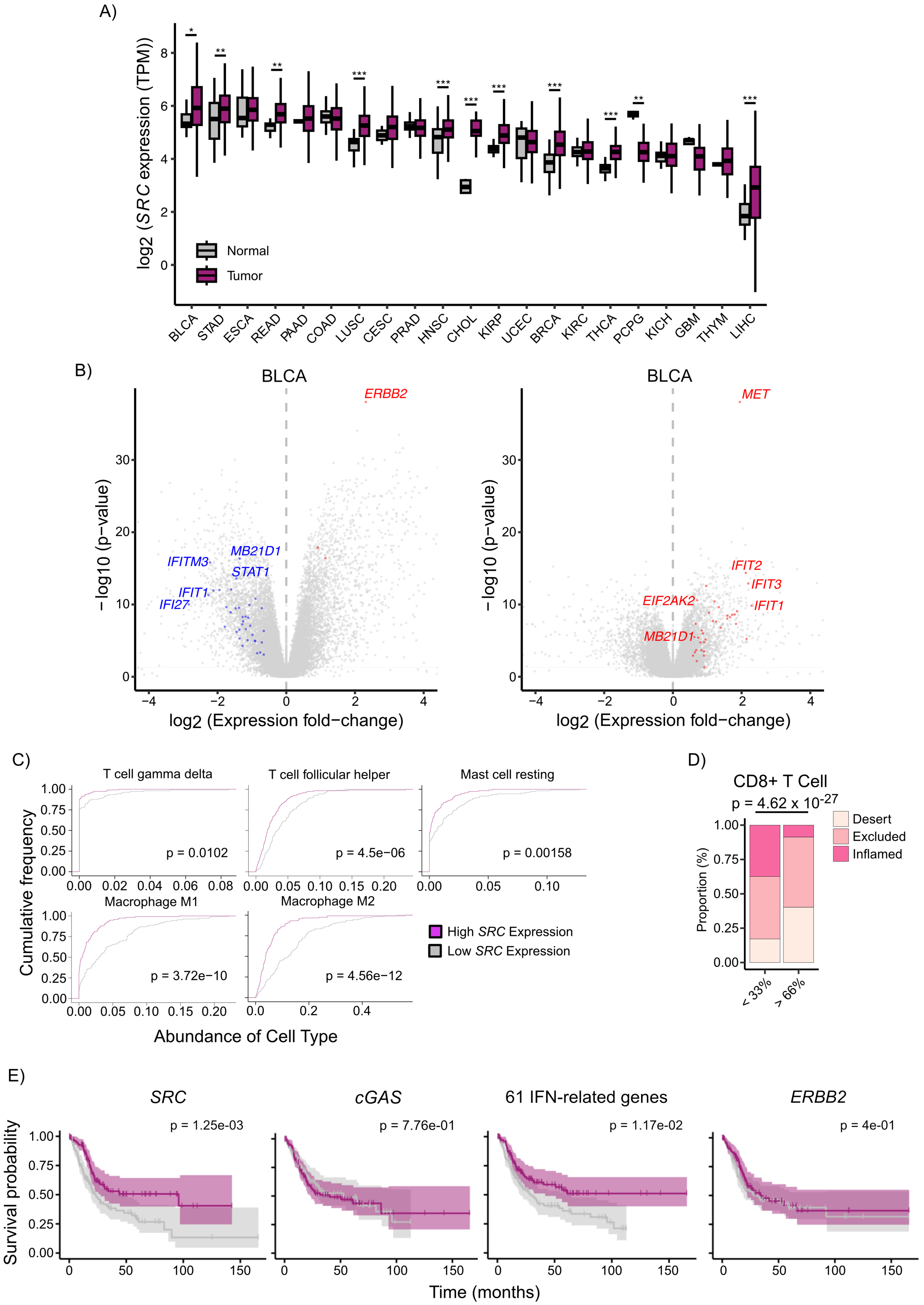
A)
log2 (SRC expression (TPM))
Normal
Tumor
BLCA
STAD
ESCA
READ
PAAD
COAD
LUSC
CESC
PRAD
HNSC
CHOL
KIRP
UCEC
BRCA
KIRC
THCA
PCPG
KICH
GBM
THYM
LIHC
B)
BLCA
ERBB2
IFITM3
MB21D1
STAT1
IFIT1
IFI27
BLCA
MET
IFIT2
IFIT3
IFIT1
EIF2AK2
MB21D1
−log10 (p−value)
log2 (Expression fold−change)
C)
T cell gamma delta
p = 0.0102
T cell follicular helper
p = 4.5e−06
Mast cell resting
p = 0.00158
Macrophage M1
p = 3.72e−10
Macrophage M2
p = 4.56e−12
High SRC Expression
Low SRC Expression
Cumulative frequency
Abundance of Cell Type
D)
CD8+ T Cell
p = 4.62 x 10-27
Desert
Excluded
Inflamed
Proportion (%)
< 33%
> 66%
E)
SRC
p = 1.25e-03
cGAS
p = 7.76e-01
61 IFN-related genes
p = 1.17e-02
ERBB2
p = 4e-01
Survival probability
Time (months)

Figure S1: *SRC* expression negatively correlates with immune response in bladder cancer. (A) *SRC* expression in primary tumors and normal counterparts from TCGA. Expression levels depicted in transcripts per million (TPM). Statistical significance determined using two-sided Mann-Whitney U-test. **(B)** *ERBB2* and *MET* correlation volcano gene expression plots in primary BLCA. Colored dots represent Type I IFN genes with significant negative (blue) or positive (red) correlation. High (upper 66th percentile) versus low (lower 33rd percentile) oncogene expressing samples were used analyzed. **(C)** CIBERSORT analysis of immune cell abundance in BLCA tumors. High (upper 66th percentile) versus low (lower 33rd percentile) *SRC* expressing samples were analyzed. Statistical significance determined using two-sided Mann-Whitney U-test. **(D)** CD8+ T cell IHC staining in advanced urothelial cancer. High (upper 66th percentile) versus low (lower 33rd percentile) SRC expressing samples were analyzed. Statistical significance determined using multinomial proportion test. **(E)** Kaplan-Meier (KM) estimation of survival of BLCA patients and correlation with *SRC, cGAS, ERBB2,* and IFN-related genes. p-value estimation from the log-rank test,

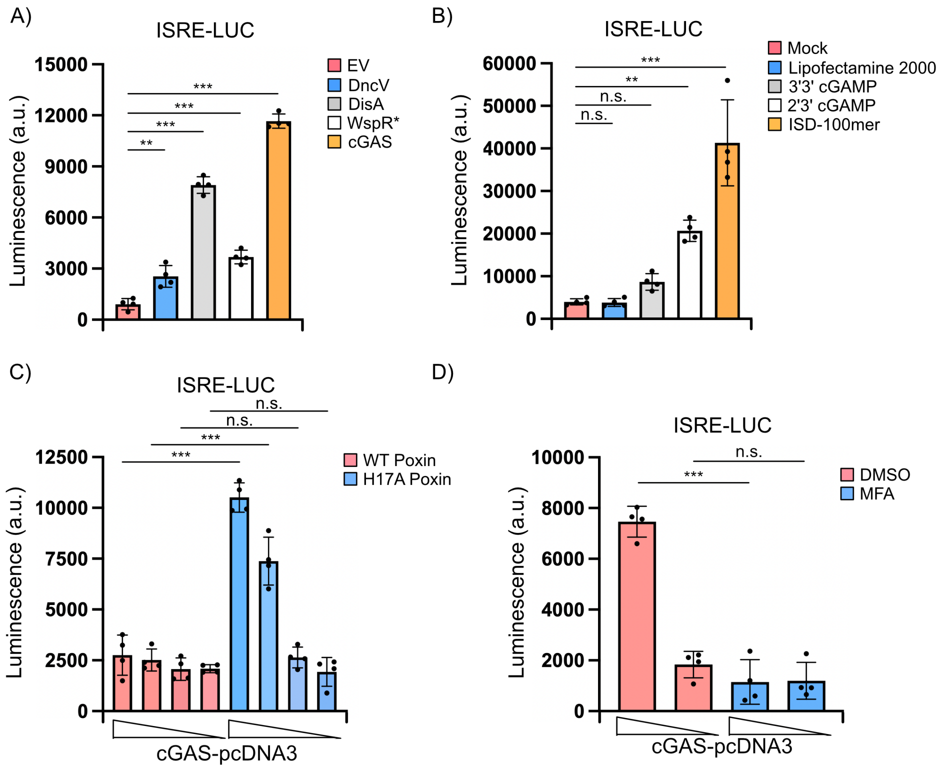

Figure S2: Cyclic dinucleotides including 2’3’ cGAMP are transferred from transfected cells into L929-ISRE-LUC reporter cells by co-culturing via gap junctions. (A) Co-culture of L929-ISRE-LUC cells with HEK293T cells transfected with pcDNA3 empty vector (EV) or CDN cyclases (n=4). **(B)** L929-ISRE-LUC cells were mock treated, mock transfected with lipofectamine 2000, or transfected with 50 µM 3’3’ cGAMP, 50 µM 2’3’ cGAMP, or 1 µg ISD-100mer. Luciferase activity was quantified 18 h later (n=4). **(C)** Co-culture of L929-ISRE-LUC cells with HEK293T cells transfected with decreasing concentrations of cGAS-pcDNA3 (1000, 100, 10, 0 ng) and 3 µg of either wildtype (WT) or catalytically inactive (H17A) Poxin (n=4). **(D)** Co-culture of L929-ISRE-LUC cells with HEK293T cells transfected with cGAS-pcDNA3 (1000 or 0 ng) in the presence or absence of the gap junction inhibitor meclofenamic acid (MFA) (100 µM) (n=4). Statistical significance tests: One-way ANOVA with Tukey test (A-D). Representative assays shown: (A-D) n=2.

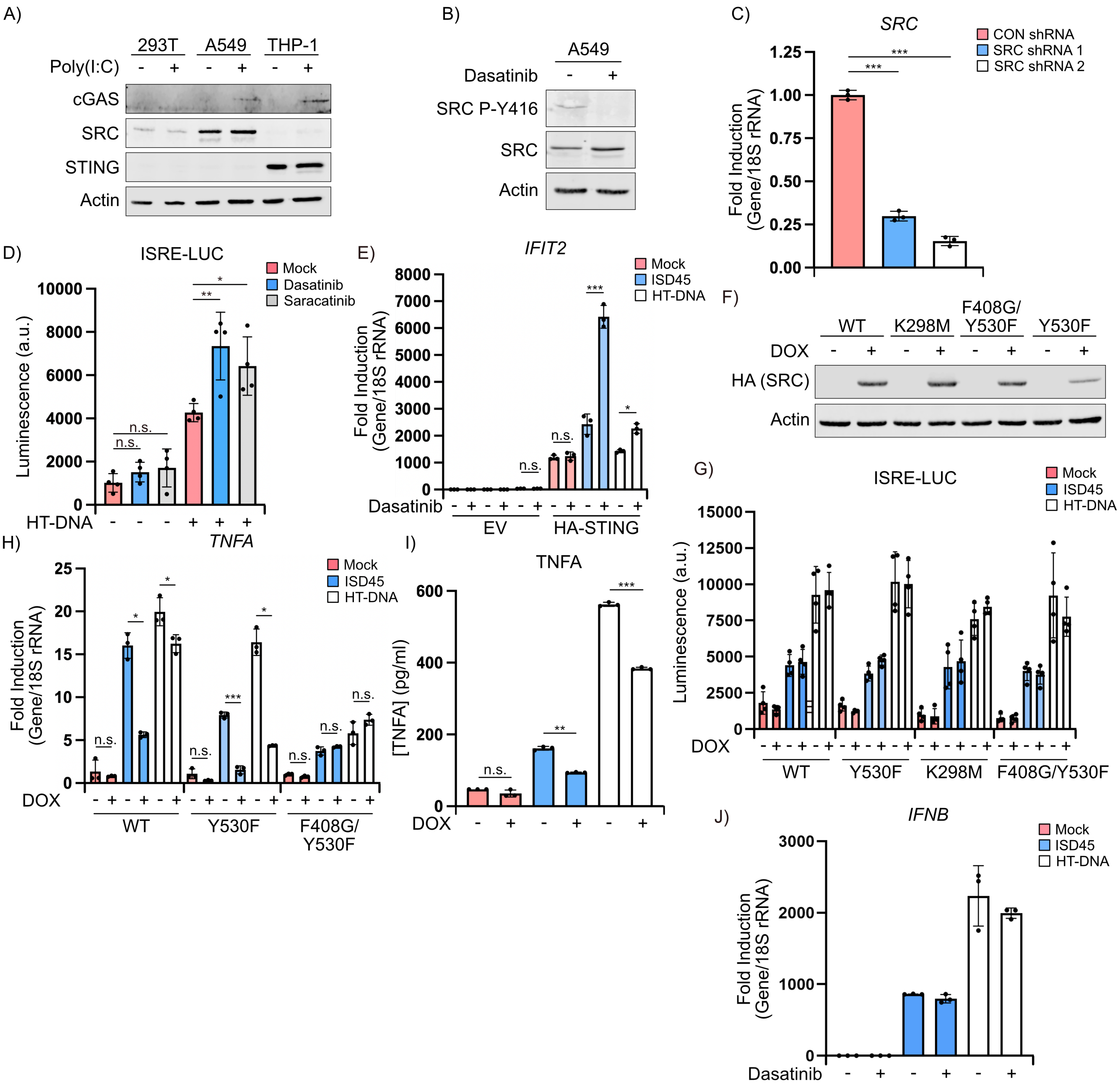
A)
293T
A549
THP-1
Poly(I:C)
cGAS
SRC
STING
Actin
B)
A549
Dasatinib
SRC P-Y416
SRC
Actin
C)
SRC
CON shRNA
SRC shRNA 1
SRC shRNA 2
Fold Induction (Gene/18S rRNA)
D)
ISRE-LUC
Mock
Dasatinib
Saracatinib
Luminescence (a.u.)
HT-DNA
E)
IFIT2
Mock
ISD45
HT-DNA
Fold Induction (Gene/18S rRNA)
Dasatinib
EV
HA-STING
F)
WT
K298M
F408G/ Y530F
Y530F
DOX
HA (SRC)
Actin
G)
ISRE-LUC
Mock
ISD45
HT-DNA
Luminescence (a.u.)
DOX
WT
Y530F
K298M
F408G/Y530F
H)
TNFA
Mock
ISD45
HT-DNA
Fold Induction (Gene/18S rRNA)
DOX
WT
Y530F
F408G/ Y530F
I)
TNFA
[TNFA] (pg/ml)
DOX
J)
IFNB
Mock
ISD45
HT-DNA
Fold Induction (Gene/18S rRNA)
Dasatinib

Figure S3: SRC and cGAS are differentially expressed in various cell lines and impacts whether SRC inhibition or overexpression regulates DNA sensing. (A) Western blot of indicated cells treated with poly(I:C) for 16 h. **(B)** Western blot of A549 cells treated with 30 nM dasatinib or vehicle for 48 h. **(C)** RT-qPCR analysis of *SRC* expression in A549 cells transduced with DOX-inducible non-target (CON) or independent *SRC*-targeting shRNAs. DOX was added for 72 h. *SRC* levels in CON were set to 1 (n=3). **(D)** Co-culture of L929-ISRE-LUC cells with A549 cells treated with dasatinib (30nM) or saracatinib (80 nM) for 48 h followed by transfection with HT-DNA for 6 h (n=4). **(E)** RT-qPCR analysis of *IFIT2* levels from A549 cells transfected with EV or HA-STING pcDNA3 and treated with dasatinib for 48 h followed by transfection with indicated DNA ligands for 6 h (n=3). **(F)** Western blot of A549 WT and mutant HA-SRC-tet-RB SB cells treated with DOX for 48 h. **(G)** Co-culture of L929-ISRE-LUC cells with cells in (F) followed by transfection with indicated DNA ligands for 6 h (n=4). **(H)** RT-qPCR analysis of *TNFA* expression in THP-1 WT and mutant HA-SRC-tet-RB SB cells treated with DOX for 48 h followed by transfection with indicated DNA ligands for 6 h (n=3). **(I)** ELISA of TNFA levels from cells in (F). Cells were stimulated with DNA ligands for 24 h (n=3). **(J)** RT-qPCR analysis of *IFNB* levels from THP-1 cells treated with dasatinib for 48 h followed by transfection with indicated DNA ligands for 6 h (n=3). All RT-qPCR samples were normalized to 18S rRNA, and Actin was used as a Western blot loading control. Statistical significance tests: Student's t test (C, E, G-J); One-way ANOVA with Tukey test (D).. Representative luciferase, qPCR, and ELISA shown: (C-E, H-J) n=2.

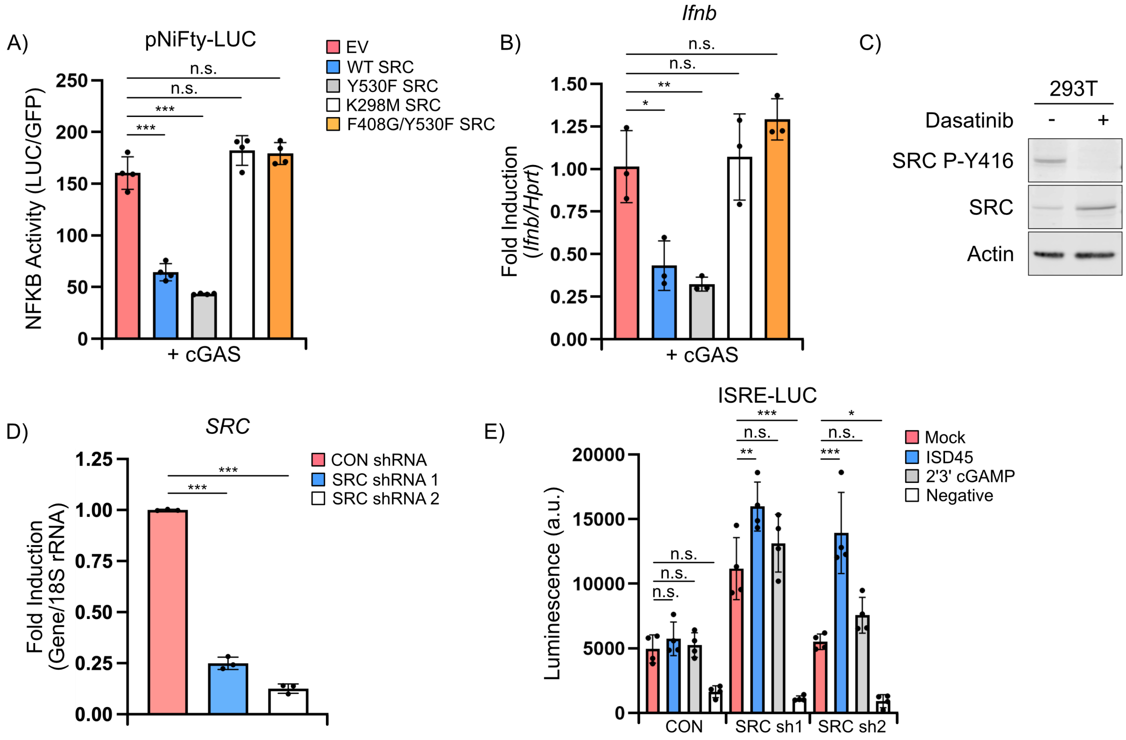

Figure S4: SRC inhibits cGAS-dependent NFKB activity and SRC depletion enhances a DNA-dependent IFN response. (A) NFKB luciferase assay from HEK293T cells transfected with pNiFty-LUC, cGAS pcDNA3, and EV or SRC-HA pcDNA3 WT or mutant pcDNA3 for 24 h. NFKB activity was analyzed by luciferase activity and normalized to transfected eGFP plasmid (n=4). **(B)** RT-qPCR analysis of murine *Ifnb* from co-cultured L929-ISRE-LUC cells in (B) (n=3). **(C)** Western blot of HEK293T cells treated with 30 nM dasatinib or vehicle for 48 h. **(D)** RT-qPCR analysis of *SRC* expression in HEK293T cells transduced with DOX-inducible non-target (CON) or independent SRC-targeting shRNAs. DOX was added for 72 h. *SRC* levels in CON were set to 1 (n=3). **(E)** Co-culture of L929-ISRE-LUC cells with cells in (D) transfected with cGAS pcDNA3 for 24 h followed by transfection of ISD45 or 2'3' cGAMP for 6 h. Cells not transfected with cGAS were used as a negative control (Negative) (n=4). All RT-qPCR samples were normalized to 18S rRNA (human) or *Hprt* (murine), and Actin was used as a Western blot loading control. Statistical significance tests: One-way ANOVA with Tukey test (A, B, D, E). Representative luciferase and qPCR shown: (A) n=3 and (B, D, E) n=2.

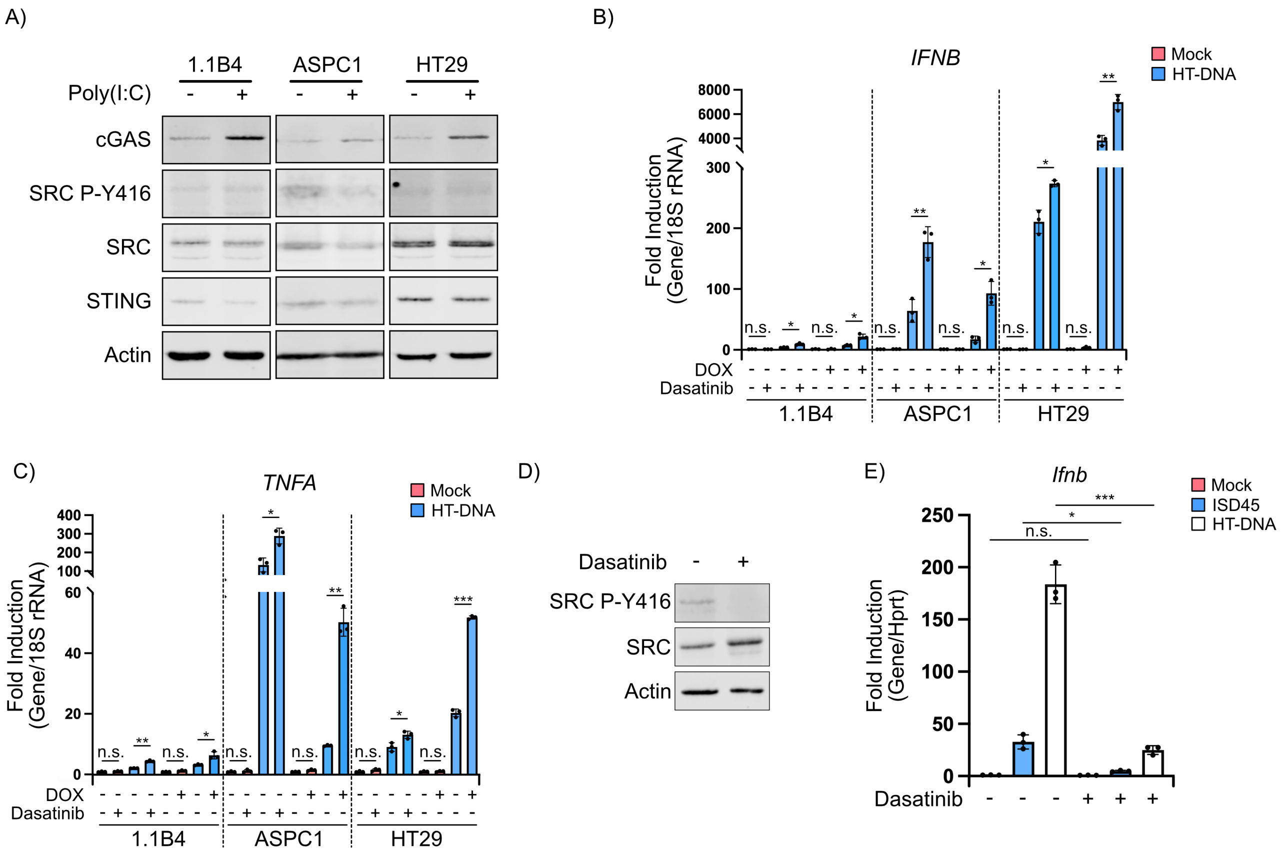

Figure S5: SRC inhibits DNA sensing in multiple human tumor lines. (A) Western blot of indicated cell lines treated with 1 μg/ml poly(I:C) for 16 h. **(B)** RT-qPCR analysis of *IFNB* expression following HT-DNA transfection for 6 h in cell lines in (A) transduced with DOX-inducible SRC-targeting shRNA or treated with 30 nM dasatinib. DOX was added for 72 h and dasatinib for 48 h. *IFNB* levels in Mock treated samples were set to 1 (n=3). **(C)** RT-qPCR of *TNFA* levels from cells in (B) (n=3). **(D)** Western blot of murine B16-BL6 cells treated with 100 nM dasatinib or vehicle for 48 h. **(E)** RT-qPCR analysis of *Ifnb* from cells in (D) treated with ISD45 or HT-DNA for 6 h (n=3). All RT-qPCR samples were normalized to 18S rRNA (human) or *Hprt* (murine), and Actin was used as a Western blot loading control. Statistical significance tests: Student's t test (B, C); One-way ANOVA with Tukey test (E). Representative qPCR shown: (B, C, E) n=2.

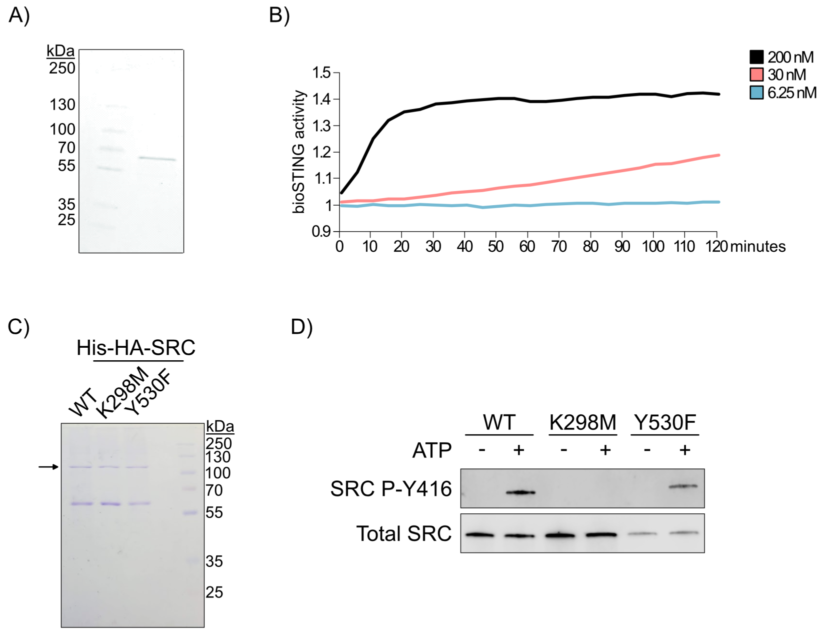

Figure S6: Human FLAG-cGAS and HA-SRC purification and activity validation. (A) Coomassie brilliant blue stain of purified FLAG-cGAS after SEC and concentration. **(B)** BioSTING FRET assay using various concentration of purified FLAG-cGAS for 2 h. **(C)** Coomassie brilliant blue stain of purified WT and mutant HA-SRC after concentration. Arrow depicts not-specific band. **(D)** Western blot of purified WT and mutant HA-SRC in vitro kinase assays using cold ATP. SRC Y416 designates autophosphorylation and SRC kinase activity.
